# Materials nanoarchitectonics at two-dimensional liquid interfaces

**DOI:** 10.3762/bjnano.10.153

**Published:** 2019-07-30

**Authors:** Katsuhiko Ariga, Michio Matsumoto, Taizo Mori, Lok Kumar Shrestha

**Affiliations:** 1 WPI Research Center for Materials Nanoarchitectonics (MANA), National Institute for Materials Science (NIMS), 1-1 Namiki, Tsukuba, Ibaraki 305-0044, Japan; 2 Graduate School of Frontier Sciences, The University of Tokyo, 5-1-5 Kashiwanoha, Kashiwa, Chiba 277-8561, Japan

**Keywords:** film, interface, low-dimensional material, nanoarchitectonics, self-assembly

## Abstract

Much attention has been paid to the synthesis of low-dimensional materials from small units such as functional molecules. Bottom-up approaches to create new low-dimensional materials with various functional units can be realized with the emerging concept of nanoarchitectonics. In this review article, we overview recent research progresses on materials nanoarchitectonics at two-dimensional liquid interfaces, which are dimensionally restricted media with some freedoms of molecular motion. Specific characteristics of molecular interactions and functions at liquid interfaces are briefly explained in the first parts. The following sections overview several topics on materials nanoarchitectonics at liquid interfaces, such as the preparation of two-dimensional metal-organic frameworks and covalent organic frameworks, and the fabrication of low-dimensional and specifically structured nanocarbons and their assemblies at liquid–liquid interfaces. Finally, interfacial nanoarchitectonics of biomaterials including the regulation of orientation and differentiation of living cells are explained. In the recent examples described in this review, various materials such as molecular machines, molecular receptors, block-copolymer, DNA origami, nanocarbon, phages, and stem cells were assembled at liquid interfaces by using various useful techniques. This review overviews techniques such as conventional Langmuir–Blodgett method, vortex Langmuir–Blodgett method, liquid–liquid interfacial precipitation, instructed assembly, and layer-by-layer assembly to give low-dimensional materials including nanowires, nanowhiskers, nanosheets, cubic objects, molecular patterns, supramolecular polymers, metal-organic frameworks and covalent organic frameworks. The nanoarchitecture materials can be used for various applications such as molecular recognition, sensors, photodetectors, supercapacitors, supramolecular differentiation, enzyme reactors, cell differentiation control, and hemodialysis.

## Review

### 1 Introduction: nanoarchitectonics for low-dimensional materials

To realize a sustainable society, there are many challenges to overcome in the next 30 years: fulfilling the needs regarding energy consumption, reducing unnecessary emissions, protecting the environment, and maximizing the efficiency of processes [1]. Various molecular technologies including chemical syntheses [2–6], ultrafine fabrications [7–11], physical analyses [12–16], materials productions [17–24], energy and environmental improvements [25–31], and biotechnological and biomedical developments [32–37] have been explored to achieve these objectives. One of the common key concepts for all these developments is regulating functional molecular systems with high spatial precision, which can often induce the efficient production, transmission, and conversion of materials, energies, and information [38]. In those examples, anisotropies in spatially defined materials or systems trigger directional and efficient flows of signals and energies. Despite many reported examples to create highly sophisticated molecular systems, there is still only a limited number of examples in which functional molecular systems are oriented or spatially confined in the bulk [39]. From these viewpoints, functional materials with low-dimensionality become a relevant part of these technologies.

**Figure 1:**
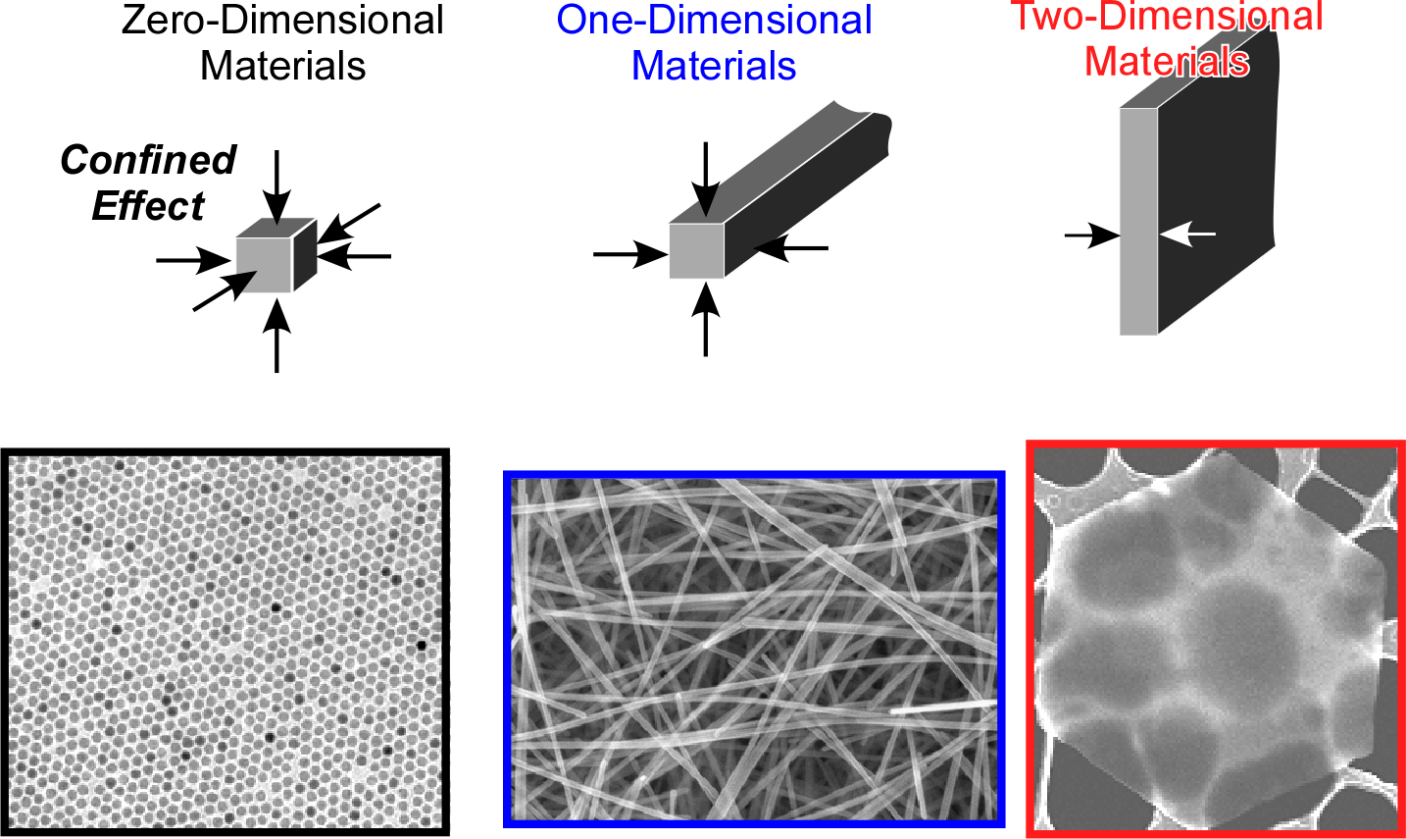
Various low-dimensional materials (zero-dimensional, one-dimensional, and two-dimensional materials) with morphology examples.

Low-dimensional materials have been extensively explored because they often exhibit unique and superior properties due to quantum effects and anisotropic effects [40–44] (Figure 1). Synthetic methodologies to yield nanoparticles and zero-dimensional materials have been developed using conventional chemical methods and/or physical perturbations such as microwave and plasma irradiation [45–51]. Carbon nanotubes, representative one-dimensional objects, were produced using catalysts as well [52–55]. Recently, two-dimensional materials such as graphene and MoS_2_ nanosheets attracted the interests of researchers because of their superior electric/electrochemical properties that make them suitable for energy and electrochemical applications [56–60]. The works include the use of two-dimensional metal oxide nanosheets for artificial photosynthesis systems, i.e., photocatalytic water splitting and fixation of carbon dioxide, which were recently reviewed by Maeda and Mallouk [61].

Despite these many intriguing demonstrations of two-dimensional materials, most of the examples reported so far utilize two-dimensional sheet materials synthesized in a top-down manner, and there is only a limited number of examples using bottom-up approaches [62]. In bottom-up approaches low-dimensional materials are constructed from small precursors such as functional molecules in order to obtain novel low-dimensional materials with various functional units [63–68]. The essential processes within these bottom-up approaches are self-assembly and self-organization based on supramolecular chemistry [69]. These supramolecular mechanisms can be widely observed in various species including small molecules, nanomaterials, and biomolecules [70–75]. Despite this generality, there are still many nontrivial fundamental challenges, which are actively studied using the quantitative analysis of self-assembly processes proposed by Hiraoka [76] and the temporal control of supramolecular polymerization by Dhiman and George [77]. Shimizu summarized various parameters regulating the self-assembly of lipid molecules for producing structurally well-regulated one-dimensional nanotubes [78]. Self-assembled and polymerized materials are often used in sensing devices utilizing molecular imprinting mechanisms [79]. Two-dimensional films made of assemblies of ion-recognizing macrocyclic host molecules, ion-exchangers, and indicative dyes were incorporated in an optode system detecting caesium ions in tap water and seawater [80]. Photo-controllable molecular devices were successfully fabricated using two-dimensional self-assembled monolayer technology as recently reviewed by Suda [81].

Hierarchic functional systems fabricated with low-dimensional materials are actively investigated. For example, Lvov and co-workers reported the immobilization of small functional materials such as metal clusters and metal catalysts within one-dimensional halloysite clay nanotubes to make them work under appropriate protection from external disturbances [82–84]. Zhong and Xu summarized, in their recent review, the preparation of metal nanoparticles for hydrogen generation from liquid chemical hydrides [85]. In their review, the usage of effective catalysts within low-dimensional cages of metal-organic frameworks was reported. Jayavel, Shrestha, and co-workers demonstrated the enhanced performance of electrochemical supercapacitors using composites of cobalt oxide nanoparticles and reduced graphene oxide, which are zero-dimensional and two-dimensional nanomaterials, respectively [86]. Leong and co-workers reported a sophisticated strategy to realize chemotherapy targeting at cancer cells using the controlled assembly and disassembly of layer-by-layer hybrid structures made of two dimensional MoS_2_ nanosheets with DNA [87].

**Figure 2:**
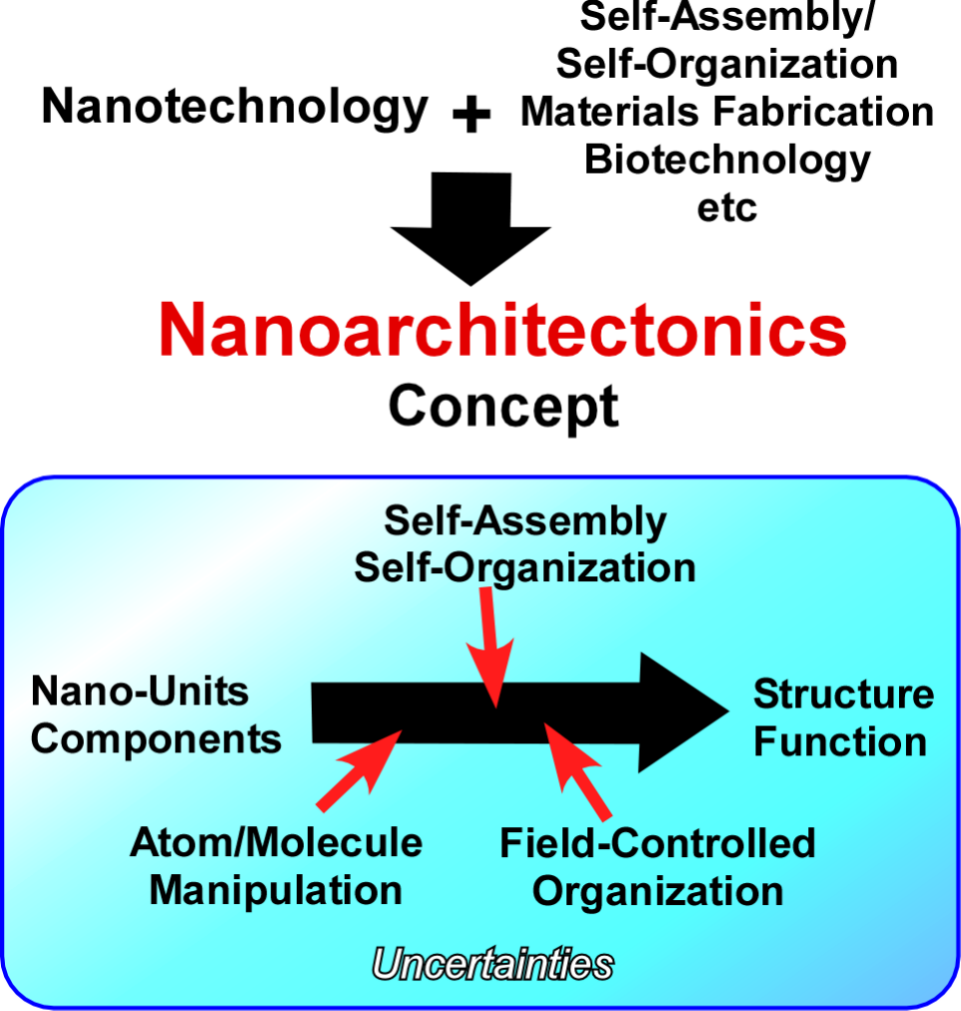
Outline of nanoarchitectonics strategies to obtain structures and functions through the manipulation of atoms and molecules, self-assembly and self-organization, and field-controlled organization.

The preparation of functional low-dimensional materials requires preservation of nanoscale features in their construction processes. This characteristic is also important in the emerging concept of nanoarchitectonics, which was initiated by Masakazu Aono in 2000 [88–91]. This concept is even regarded as the next step of nanotechnology combining various research disciplines such as organic synthesis, physical materials control, supramolecular chemistry, and biology [92–94]. In this concept, materials and systems can be engineered through the manipulation of atoms and molecules, self-assembly and self-organization, and field-controlled organization (Figure 2). Unlike the well-established microfabrication and other techniques at microscopic and macroscopic levels, the nanoarchitectonics procedures have to take into account several uncertainties such as thermal fluctuations, quantum effects, and uncontrolled mutual interactions at the nanoscale [95–96].

Because of its general applicability, research approaches with the nanoarchitectonics concept are now seen in many recent publications in various fields including materials production [97–98], structural fabrication [99–108], sensing [109–112], energy applications [113–117], environmental protection [118–119], catalysts [120–121], biology [122–124], and biomedical applications [125–126]. For example, the following recent research works on low-dimensional materials have been carried out using the nanoarchitectonics concept: Hasegawa and co-workers used atom-manipulation nanoarchitectonics (controlled single atom/ion transfer) to regulate the number of dopant atoms in one-dimensional solid electrolyte nanodots (α-Ag_2+δ_S) [127]. The nanoarchitectonic construction of one-dimensional nanowires from II–VI semiconductors was demonstrated for the use as wavelength division multiplexer as reported by Yan, Zhao and co-workers [128]. Other one-dimensional functional structures such as porphyrin-functionalized DNA (by Stulz [129]), DNA-based complex structures for ultrasensitive mercury detection (by Govindaraju and co-workers [130]), self-assembled chiral twisted and helical nanofibers (by Liu and co-workers [131]), and supramolecular assemblies with short peptides and their bio-functions (by Yan and co-workers [132]) have been investigated. As examples of research efforts regarding two-dimensional nanoarchitectures, the enhanced reduction of nitrogen oxides by facet-engineered two-dimensional CuO petal assemblies (by Abe and co-workers [133]), perovskite nanosheets and their layer-by-layer assemblies as high-*k* dielectric/ferroelectric materials (by Osada and Sasaki [134]), the manipulation of transition-metal dichalcogenides nanosheets for the usage in energy storage/conversion applications (by Xu, Lee, and co-workers [135]) and substrate channelling between enzymes with graphene oxide nanosheets (by Yang and co-workers [136]) can be mentioned.

The nanoarchitectonics bottom-up approaches preserving the nanostructural properties are highly useful for the fabrication of low-dimensional materials and the subsequent construction of functional structures from low-dimensional materials. Especially, nanoarchitectonics fabrication in motional restricted and dimensionally confined media would be beneficial for the production of low-dimensional materials. Therefore, in this review article, we overview recent research progresses on materials nanoarchitectonics at two-dimensional liquid interfaces, which are dimensionally restricted media with certain degrees of motional freedom [137–138]. In the next section, specific features of molecular interactions and functions at liquid interfaces, as well as two-dimensional molecular patterning, are briefly explained. In the following sections, several topics of materials nanoarchitectonics at liquid interfaces such as the preparation of two-dimensional metal-organic frameworks (MOFs) and covalent organic frameworks (COFs), the fabrication of multi-dimensionally structured nanocarbons and their assemblies, and the interfacial nanoarchitectonics of biomaterials are exemplified.

### 2 Unique features of liquid interfaces and formation of two-dimensional patterns

#### 2.1 Unique features of liquid interfaces

Gas–liquid interfaces and liquid–liquid interfaces are categorized as interfacial environments with certain degrees of freedom of molecular mobility (dynamism). These interfaces with liquids have several intrinsic features: (i) They are environments of two different phases; (ii) they exhibit a discontinuous change of the dielectric constant; (iii) they are highly directional environments restricting molecular motion only in the vertical direction. These features create several unique features that are described in the following.

Interfaces are generally formed by two immiscible phases. In many research examples utilizing liquid–liquid interfaces, the immiscible liquids dissolve different species that can only come into contact at the interface. At gas–liquid interfaces, insoluble components remain only at the interface and interact with other molecules (or materials) diffused from the underneath liquid phase. These circumstances can induce the generation of low-dimensional materials. In addition, physics and chemistry of molecular interactions at liquid interfaces are significantly different from those observed in homogeneous solutions [139–141].

**Figure 3:**
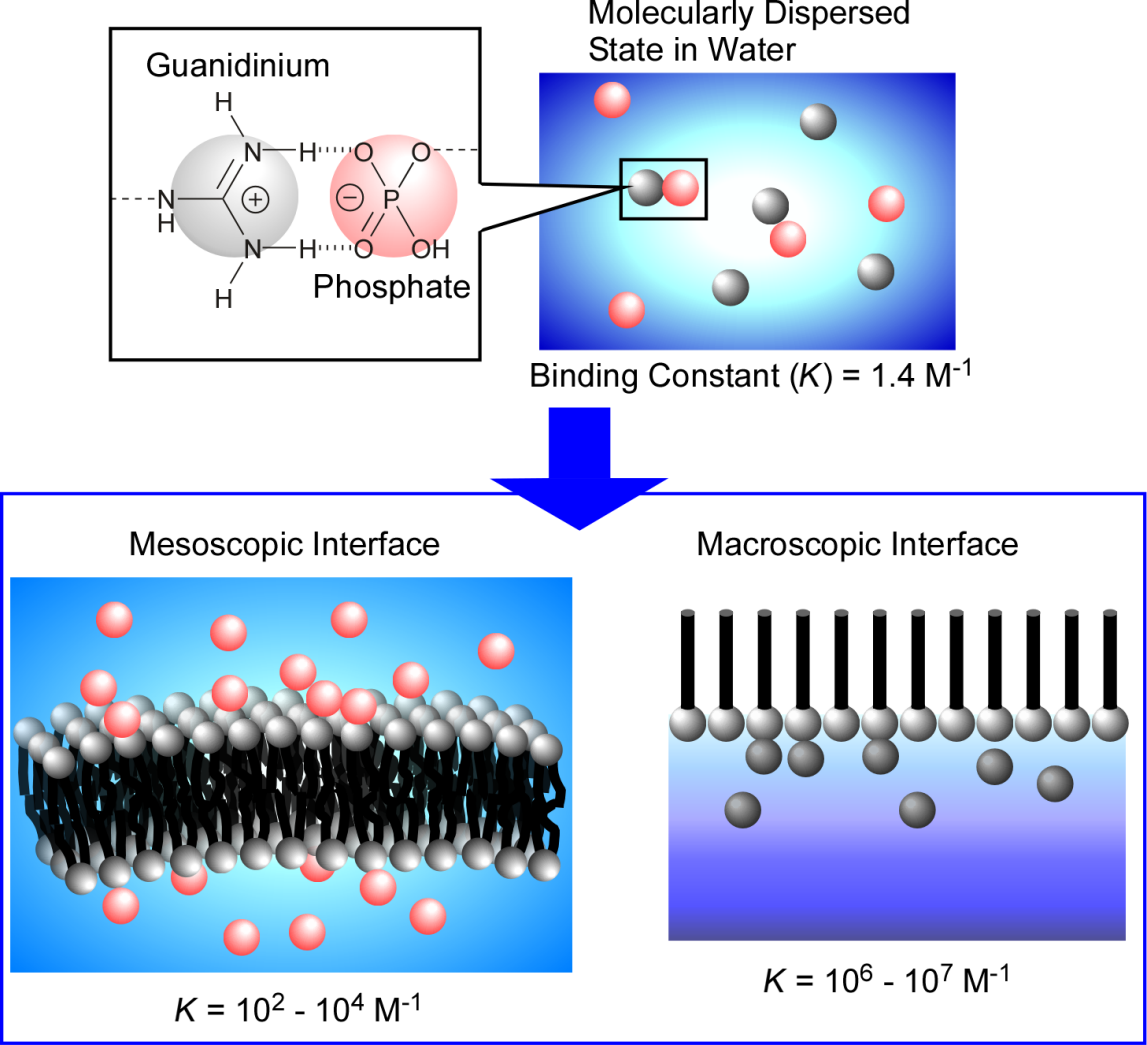
Systematic studies on binding constants for phosphate and guanidinium in different aqueous environments: 1.4 M^−1^ dispersed in water; 10^2^ to 10^4^ M^−1^ at disorganized mesoscopic interfaces; 10^6^ to 10^7^ M^−1^ at the macroscopic air–water interface. Adapted with permission from [144], copyright 2019 American Chemical Society.

Unique features of molecular interactions can be clearly observed at the air–water interface [142–144]. Although molecular recognition via hydrogen bonding are quite difficult in a highly polar aqueous media, the molecular recognition of sugars [145–146], peptides [147–149], amino acids [150], nucleic acid bases [151–152], and nucleotides [153–154] is accomplishable at the air–water interface even though this recognition relies on hydrogen bonding. Systematic studies on binding constants of a fixed recognition pair, phosphate and guanidinium ions, revealed a significant influence of the interfacial environment on the interactions between the molecules embedded at various interfaces (Figure 3) [155]. The binding constant between phosphate and guanidinium ions dispersed in water was reported as 1.4 M^−1^ [156]. Altering the recognition media to rather disorganized mesoscopic interfaces drastically increases the binding constants. The binding constants of phosphate derivatives to the surfaces of guanidinium-functionalized aqueous micelles and lipid bilayers reaches values of 10^2^ to 10^4^ M^−1^. Surprisingly, the binding constant of the same recognition pair further increases to 10^6^ to 10^7^ M^−1^ when a macroscopic less dynamic interface, the air–water interface, is used as the recognition medium [157–158]. Similarly, strongly enhanced binding constants were commonly observed at the air–water interface for various recognition pairs.

**Figure 4:**
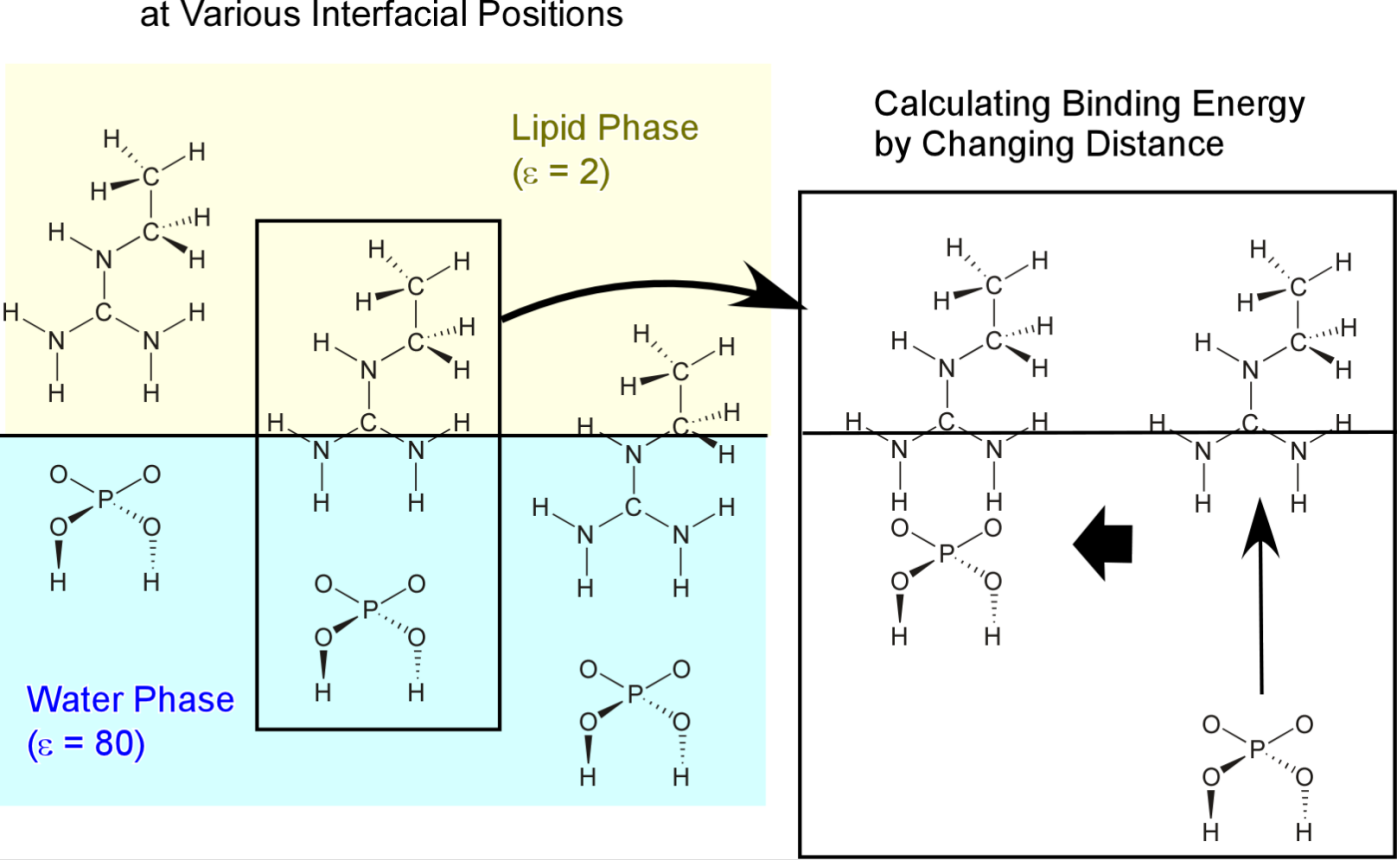
A model for calculations of the binding constant between guanidinium and phosphate at the air–water interface where the pair structures of phosphate and guanidinium are placed in a model of two phases with different dielectric constants of 2 (lipid phase) and 80 (water phase). The binding energy and binding constant was calculated while changing distance between guanidinium and phosphate.

Mechanisms enhancing the molecular interaction at the air–water interface were investigated through quantum chemical approaches [159–161]. As simply illustrated in Figure 4, simplified recognition-pair structures of phosphate and guanidinium were placed at a model interface of two phases with different dielectric constants of 2 (lipid phase) and 80 (water phase). By fixing the position of the guanidinium moiety at the interface and changing the relative location of the phosphate functional group, the recognition energy was monitored as a function of the relative location. The most stable relative distance was estimated from the energy minimum in the energy diagram, and the binding energies and binding constants were calculated at those interfacial positions. A series of calculations revealed that large binding constants can be obtained when the binding site locates in the phase of the lower dielectric constant. In contrast, when the binding site was located to be deep in the phase of the higher dielectric constant, the binding constants were calculated to decrease significantly. Interestingly, sufficiently high binding constants were confirmed even when the hydrogen bonding sites were exposed to the high-dielectric medium at the very vicinity of the low-dielectric medium. These simulations hint at the mechanism of enhanced molecular recognition at these interfaces. The non-polar phase greatly contributes to enhance molecular recognition.

These facts may answer the question of biological molecular recognition in aqueous media, in which hydrogen bonding plays an essential role in realizing those highly sophisticated systems [162]. Molecular recognitions in biological systems occur mostly at interfaces including cell membrane surfaces, inner surfaces of receptor pockets in enzymes, and macromolecular interfaces of DNA. We expect materials nanoarchitectonics with features of enhanced molecular interactions to create low-dimensional materials at interfaces of two phases with different dielectric natures.

Another distinctive characteristic of liquid interfaces is the anisotropic environment regarding molecular motion. Certain degrees of motional freedom exist along the interfacial plane, which can be deformed at the macroscopic level (compression, expansion, and bending). In contrast, molecular motion is virtually inhibited in the vertical direction. Therefore, two significantly different scales of motion, macroscopic lateral motion and nanoscopic vertical motion, are connected at liquid interfaces [163–166]. For example, motion and function of molecular machines and molecular receptors in monolayers at the air–water interface can be controlled by macroscopic lateral motion such as mechanical compression and expansion of the monolayers. Macroscopic mechanical deformation of the interfacial media at the scale of centimetres or metres can regulate nanometre-scale conformational changes of the molecular machines, for instance, to capture and release guest molecules [167–168], to rotate of molecular rotors [169–170], to open and close molecular pliers [171–172], or in indicator displacement assays of glucose based on fluorescence resonance energy transfer [173]. Subtle conformational changes of molecular receptors at the air–water interface results in a change of the chiral selectivity towards aqueous amino acids [174–175], or of the optimum guest structure from thymine to uracil derivatives [176–177]. Regulation of molecular interaction at liquid interfaces yields a novel concept for the molecular tuning of functions [178–180]. This is a new concept beyond the following well-known important concepts: the 1st generation of molecular recognition at the most stable state (basics for supramolecular chemistry, Nobel prize in 1987 [181–183]); the 2nd generation molecular controls based on external stimuli switching [184–188] (basics for molecular machines, Nobel prize in 2016 [189–191]). The anisotropic dynamics at liquid interfaces described above are expected to play a crucial role in the production of low-dimensional materials and systems.

#### 2.2 Two-dimensional molecular patterning and production of low-dimensional materials

**Figure 5:**
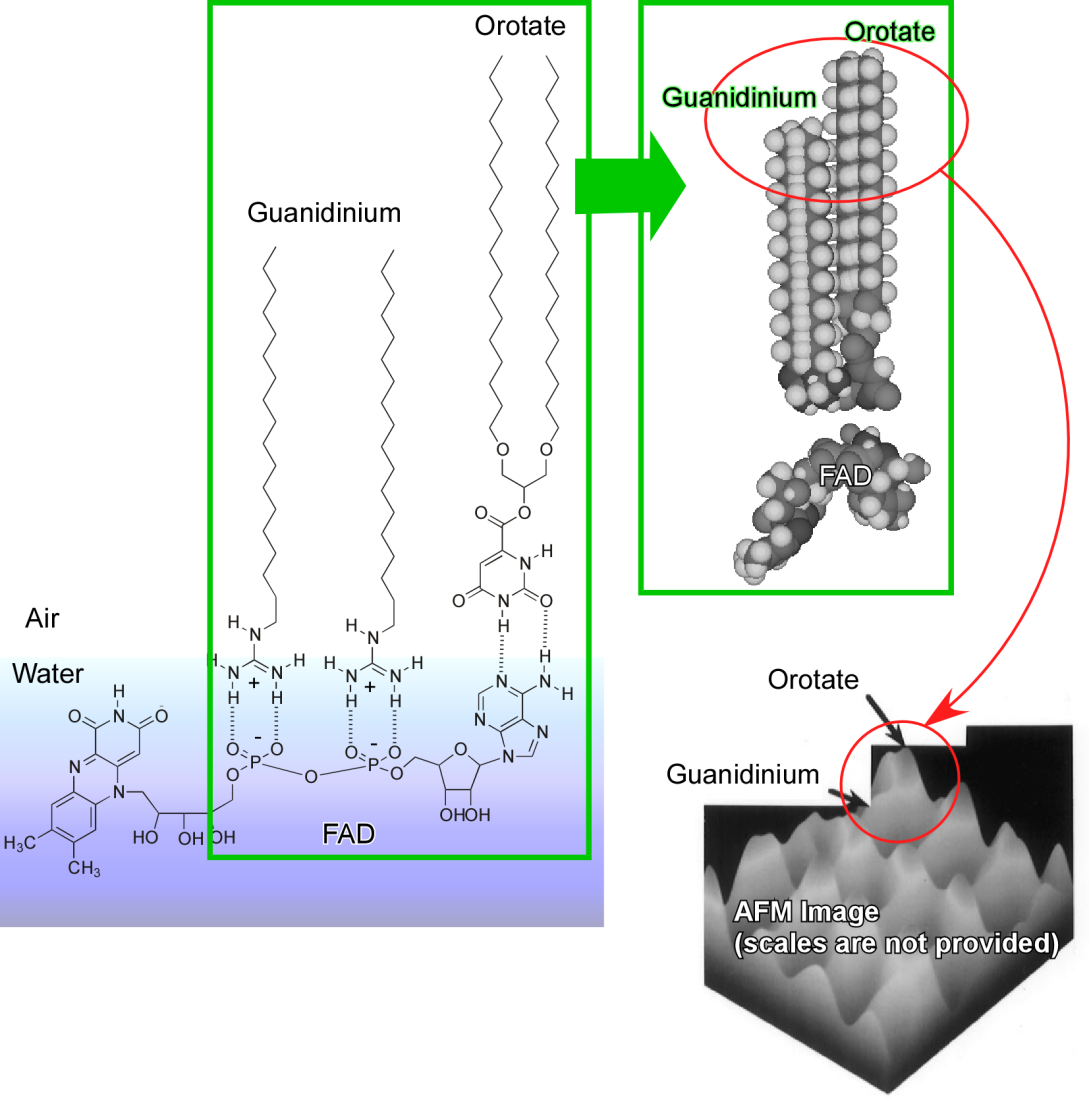
Formation of two-dimensional patterned structures using flavin adenine dinucleotide (FAD), which can bind site-specifically to two monolayer components, a guanidinium lipid or an orotate lipid, with the phosphate moieties and adenosine part, respectively. Adapted with permission from [195], copyright 1997 American Chemical Society.

Enhanced molecular interaction and two-dimensionally confined motion at liquid interfaces are advantageous for the fabrication of two-dimensional patterned structures with high structural precision [192–193]. In the case exemplified in Figure 5, flavin adenine dinucleotide (FAD) was dissolved in an aqueous subphase [194–195]. FAD can bind specifically to two monolayer components, a guanidinium lipid or an orotate lipid. These molecules bind site-specifically to the phosphate moieties or the adenosine part, respectively. Lateral compression of the complexed monolayer finally results in two-dimensional regular molecular patterns. The difference between the molecular lengths of the guanidinium/phosphate and orotate/adenosine pairs yields regular dip patterns with sub-nanometre precision. Similar methodologies, i.e., crystallinity controlled two-dimensional patterns based on guanidinium/carboxylate molecular recognition [196] and the two-dimensional assembly of one-dimensional supramolecular polymers formed between alkylated melamine and aqueous barbiturate [197] have been also accomplished.

Oishi and co-workers utilized the balance between two competitive interactions, the phase separation of fluorocarbons and hydrocarbons and the attractive interaction between guanidiuium and carboxylate, to fabricate nanoscopic domains within a two-dimensional mixture of hydrocarbon guanidinium and fluorocarbon carboxylic acid [198]. The domain sizes can be tuned by altering the mixing ratio of the two components. In a recent review article by Krafft and co-workers [199], the formation of surface nanodomains and their hierarchical organization with higher complexity with semi-fluorinated alkanes and related molecules (molecules consisting of two blocks, a fluorocarbon block and a hydrocarbon block, in a single chain) at the air–water interface is discussed. They expect possible applications of these two-dimensional nanodomains in sensors, nanoelectronics and nanophotonics.

Pellerin, Bazuin, and co-workers investigated the mechanisms of formation and transformation of zero-dimensional structures within two-dimensional media (dot-dispersed monolayers of block copolymers) [200]. Self-assembled monolayers of polystyrene-*b*-poly(4-vinylpyridine) and its supramolecular complex with 3-*n*-pentadecylphenol at the air–water interface alter their assembly patterns from hexagonal to squared upon applying lateral pressure. The transition is caused by the entropically driven molecular folding of the poly(4-vinylpyridine) moieties, in which the polymer transforms from a two-dimensional motif to a three-dimensional motif. The proposed mechanism might be generalized for zero-dimensional dot-dispersed monolayers of block-copolymers. Wen and co-workers reported the drastic modification of two-dimensionally patterned Langmuir–Blodgett (LB) films of polystyrene-*b*-poly(2-vinylpyridine) transferred from the air–water interface through acetone vapour annealing [201]. Complicated morphology shifts such as swelling, coalescing of aggregates, bicontinuous pattern formation, one-dimensional droplet formation, and the periodic evolution of the droplets were observed.

**Figure 6:**
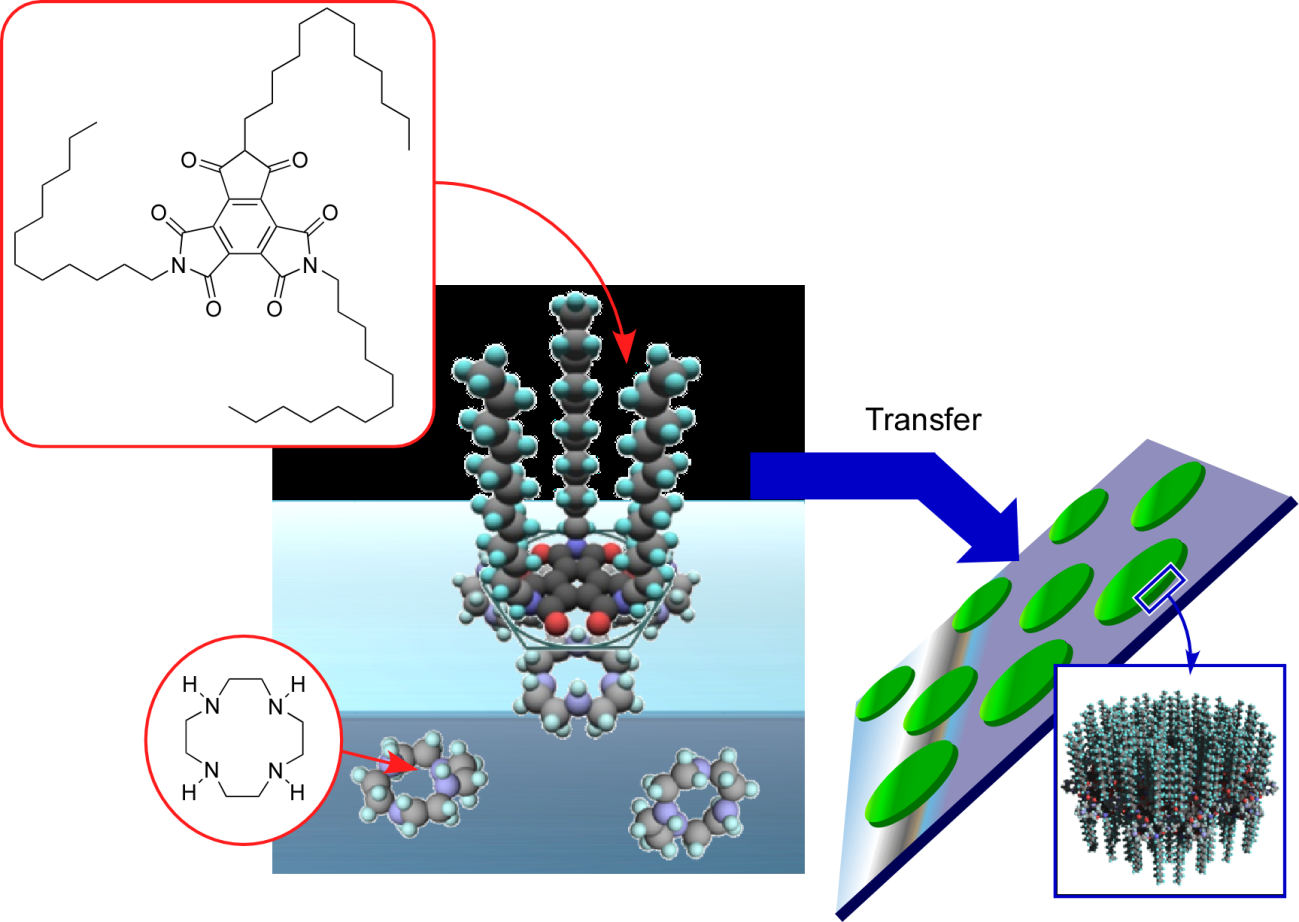
Formation of two-dimensional arrays of nanodisk-like assemblies via touching transfer of a monolayer of amphiphilic tri-*n*-dodecylmellitic triimide with three alkyl chains as a monolayer component and 1,4,7,10-tetraazacyclododecane (cyclen) as a subphase template. Adapted from [75], copyright 2019 The Authors.

Mori et al. reported the formation of two-dimensional arrays of disk-shaped nano-assemblies at the air–water interface yielding a monolayer that was successively transferred onto solid surfaces via the contacting method (Figure 6) [202]. Unlike two-dimensional molecular patterning though molecular recognition, which has been described in the previous parts, a rather ambiguous interaction between amphiphilic triimide with three alkyl chains, a monolayer component, and 1,4,7,10-tetraazacyclododecane (cyclen), a subphase template, was used to regulate the formation of hydrogen bonds between the imide functional groups as hydrogen-bond acceptors and the secondary amine moieties of cyclen as hydrogen-bond donors. The two-dimensional quick dewetting process on a Langmuir–Schaefer-type surface can induce a good dispersion of nanodisks. Although the heights of nanodisks reported so far are within a narrow range between 2.6 and 2.9 nm, their diameters can widely range from 46 to 73 nm depending on their lateral surface pressure at the air–water interface. The fabricated array structures of nanodisks can be also transferred to metal surfaces such as platinum surfaces. The examples demonstrated that the combination of rather ambiguous molecular interactions and transfer processes can create precise patterns, which are one of the main concept of nanoarchitectonics.

**Figure 7:**
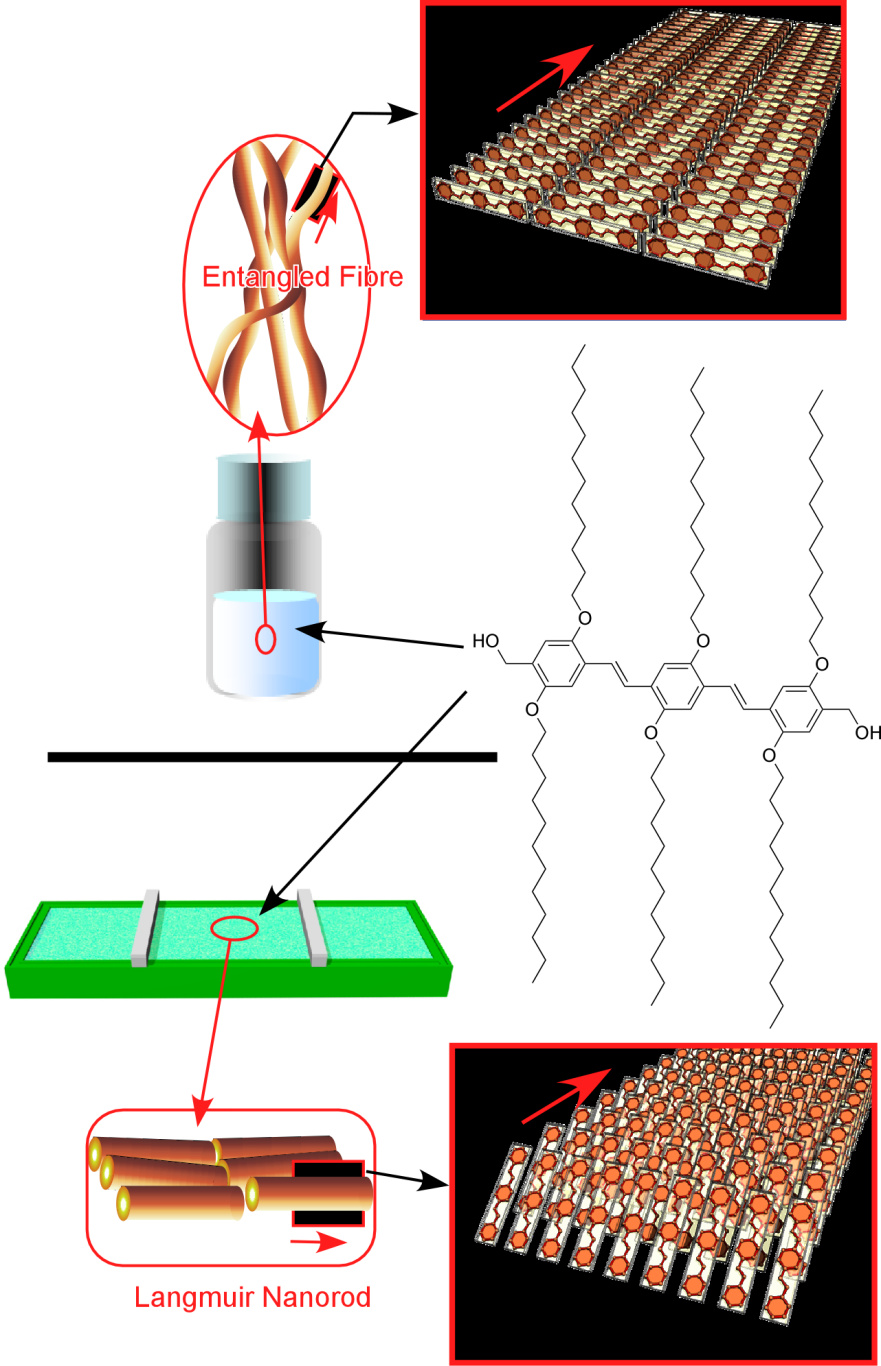
Difference of one-dimensional assemblies of an oligo(*p*-phenylenevinylene) derivative formed in different media: (top) entangled fibre structures formed in toluene solution and drop-cast on a solid surface; (bottom) aligned rods formed at the air–water interface. Adapted from [75], copyright 2019 The Authors.

Small gelation molecules often form one-dimensional assembled structures [203–207]. The inner structures of these one-dimensional objects can be significantly altered through the surrounding media such as organic solutions and the air–water interface. Sakakibara et al. investigated the morphological change of one-dimensional assemblies of oligo(*p*-phenylenevinylene) induced by different media (Figure 7) [208]. In entangled fibre structures, formed in toluene solution and successively transferred on a solid surface by drop-casting, the long axis of oligo(*p*-phenylenevinylene) molecules is arranged perpendicularly to the substrate. Intra-fiber energy transfer efficiently occurs in the entangled nanofibers. Long-range excitation energy transfers are advantageous for excitation energy transfer. In contrast, the oligo(*p*-phenylenevinylene) molecular units are oriented in parallel to the long axis of the aligned rods that were formed at the air–water interface from its homogeneous solution in chloroform. The excitation preferences between inter- and intra-fiber can be altered by controlling the arrangement of the aligned rods. In environments of closely packed nanorods (when the inter-rod distance was less than ca. 70 nm), enhanced excitation transfer was observed, indicating that fluorescence would be efficiently enhanced within well-aligned nanorods prepared at the air–water interface.

Inorganic low-dimensional nanomaterials often exhibit interesting properties [209–215]. Such materials can be also nano-engineered at liquid interfaces. Niederberger and co-workers successfully fabricated two-dimensionally aligned arrays of one-dimensional W_18_O_49_ nanowires and used them for H_2_-sensing at room temperature [216]. The diameters of the used nanowires are less than 2 nm and their aspect ratios exceed 100. The synthesized nanowires are dispersible in organic solvents and can be fabricated in large-area aligned arrays at the air–water interface. The films were transferred onto Si/SiO_2_ substrates patterned with platinum interdigitated electrodes. An excellent sensor capability for H_2_ gas in humid air at room temperature was observed for a film of 10 layers of the aligned one-dimensional W_18_O_49_ nanowires. Various additional techniques to fabricate two-dimensional structures have been proposed. Advincula and co-workers demonstrated two-dimensional co-patterned structures of carbazole-based conductive polymers and gold by nanosphere lithography [217]. Huang and co-workers proposed a high-yield LB method for nanoparticle films through electrospray techniques to significantly reduce the spreading of droplets and used a subphase-miscible solvent [218]. The modified method may become a powerful method to fabricate two-dimensional thin films of zero-dimensional nanoparticles at liquid interface.

**Figure 8:**
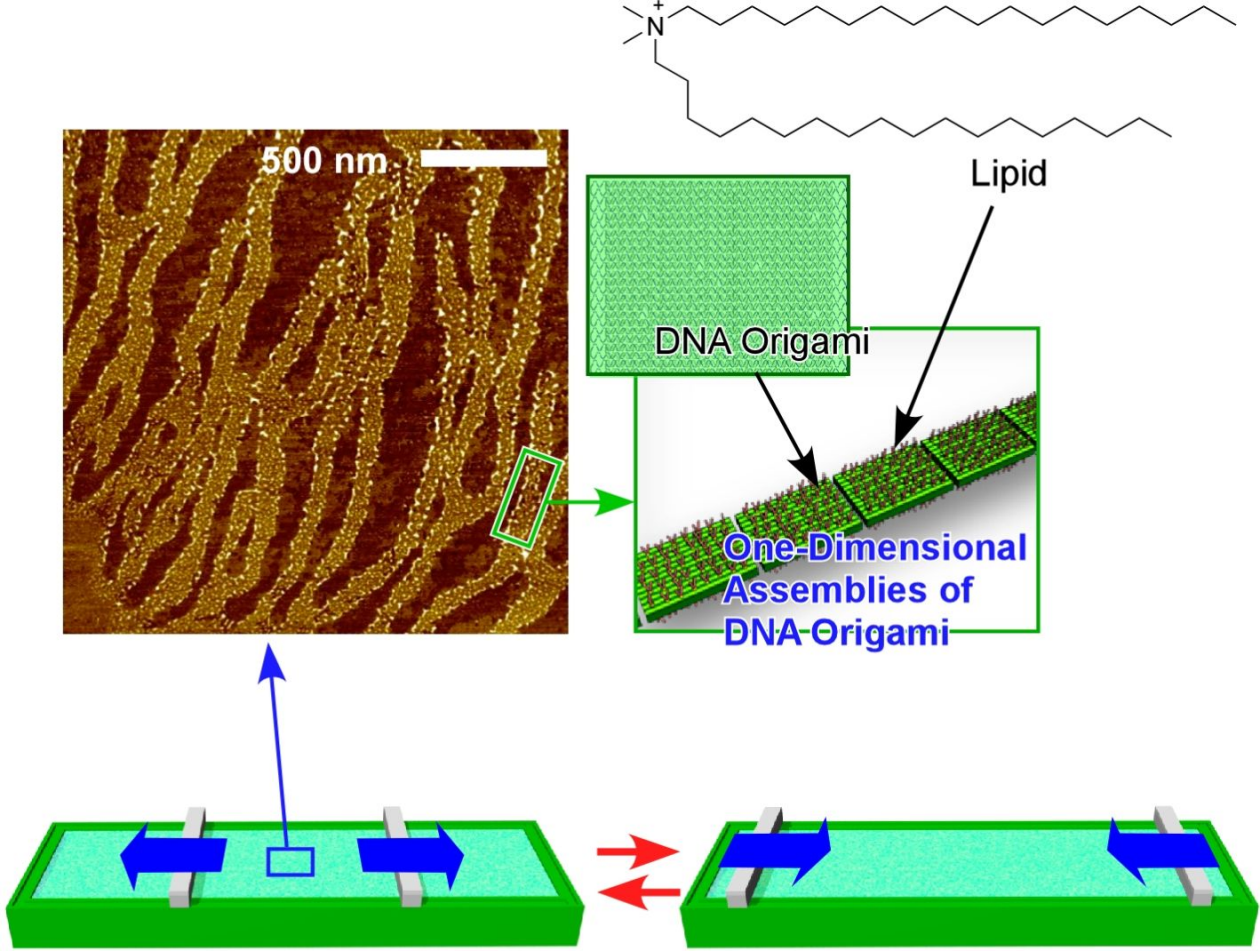
Formation of one-dimensional supramolecular polymerization of DNA origami pieces upon repeated mechanical compressions and expansions of two-dimensional air-water interface. An AFM image was reprinted with permission from [144], copyright 2019 American Chemical Society.

The lateral degree of motional freedom of the liquid interfaces can promote associations of molecules and materials for the fabrication of two-dimensionally structures. As depicted in Figure 8, Yonamine et al. successfully demonstrated the one-dimensional supramolecular polymerization of DNA origami pieces upon repeated mechanical compression and expansion of the two-dimensional air–water interface [219]. The used DNA origami pieces had a rectangle shape with 90 × 65 nm^2^, according to theoretical calculations, and were complexed with counter-cationic lipids to be soluble in organic solvents. The resulting organic solution of the DNA origami pieces was then spread on the air–water interface to form a Langmuir monolayer. Although the spread DNA origami pieces initially remained in the monomer form, the repeated mechanical compression and expansion of the Langmuir monolayer induced the interconnection of the rectangle pieces into one-dimensional polymer motifs. The origami–origami connections were formed only at the shorter sides of the rectangle pieces where dangling DNA chains remained. The enhanced capability of hydrogen-bond formation at the air–water interface resulted in one-dimensional supramolecular polymers through inter-piece connections at the specific sides. Interestingly, dynamic motion is indispensable for the formation of these supramolecular polymers of DNA origami. A simple application of high pressure is not enough to obtain supramolecular polymerization of DNA origami pieces at the air–water interface.

### 3 Interfacial nanoarchitectonics for MOF and COF

Interfaces are attractive platforms to synthesize two-dimensional materials. The recent developments of synthetic two-dimensional crystalline polymers (2DCPs), such as two-dimensional metal-organic frameworks (MOFs) and two-dimensional covalent organic frameworks (COFs), have unveiled their intriguing chemistry and properties, and have shown their potential for wide-ranging applications, such as electronics, sensing, catalysis, separation, and energy storage and conversion. However, most reported two-dimensional MOFs and COFs have been synthesised as powders, which are not easily processed into more useful forms due to their nature as cross-linked polymers. Thus, their adaption for technological applications is still challenging. Recently, liquid interfaces have been considered to be useful platforms to form thin 2DCP films, and the number of examples showing interfacially grown 2DCP films for potential applications is increasing [220].

**Figure 9:**
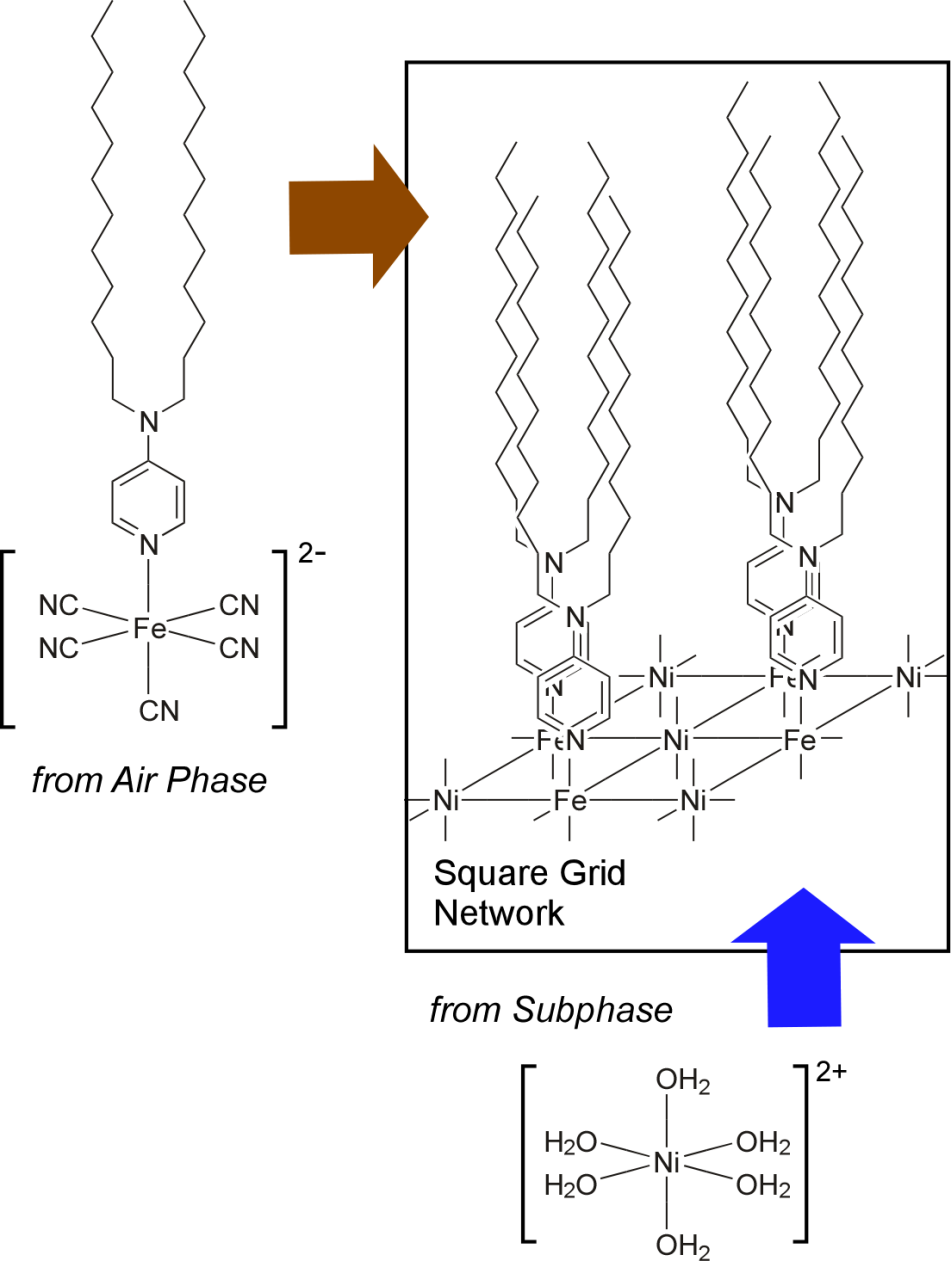
Formation of a two-dimensional iron–nickel cyanide-bridged network at the air–water interface through reaction of a Langmuir monolayer of an amphiphilic pentacyanoferrate complex with Ni^2+^ ions from the subphase.

The air–water interface is the most commonly used liquid interface to grow 2DCP films. In 2002, Culp et al. reported a reaction of a Langmuir monolayer of an amphiphilic pentacyanoferrate complex with Ni^2+^ ions from the subphase (Figure 9) [221]. This reaction resulted in the formation of a two-dimensional iron–nickel cyanide-bridged network at the air–water interface. A small amount of the amphiphilic pentacyanoferrate complex monomer was spread from a chloroform solution to form a monolayer in a LB trough, and was subsequently connected by introducing an aqueous solution of nickel nitrate into the water phase, yielding a monolayer sheet of the two-dimensional nickel–iron cyanide grid network. Characterizations of the extended network by X-ray photoelectron spectroscopy (XPS), FTIR spectroscopy, SQUID magnetometry, X-ray absorption fine structure (XAFS), and grazing incidence synchrotron X-ray diffraction (GIXD) revealed a face-centred square grid structure with an average domain size of 3600 Å^2^.

**Figure 10:**
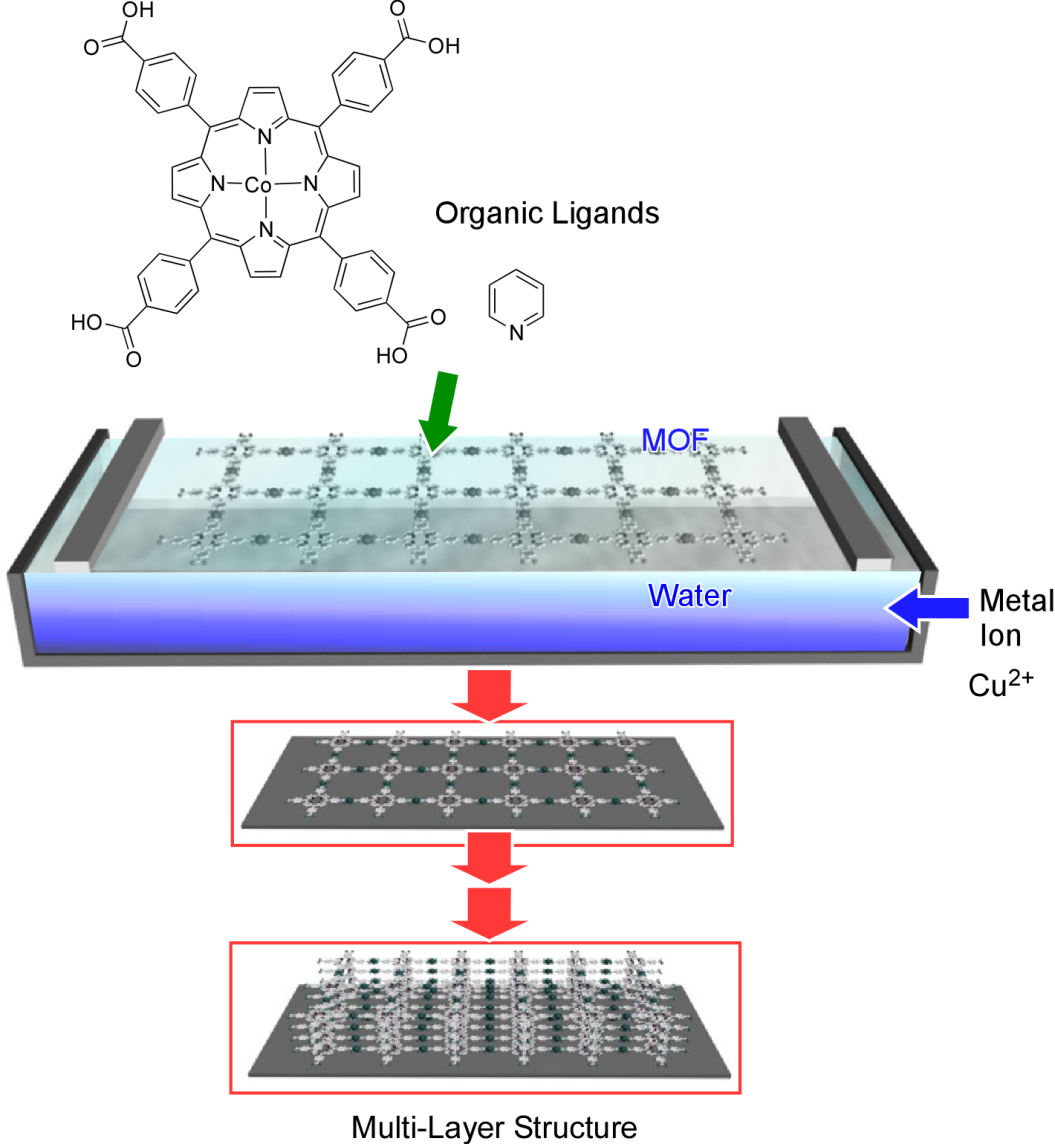
Fabrication of a multilayer of oriented porphyrin-based MOF films by repeatingly transferring and washing interfacially grown MOF layers.

Makiura et al. employed a similar method to form multilayers of an oriented porphyrin-based MOF film on top of substrates by repeating transfer and washing of interfacially grown MOF layers (Figure 10) [222]. Interestingly, the proposed structural model incorporates metal-coordinated pyridine molecules projected from the two-dimensional sheets that allow each further layer to dock in a highly ordered interdigitated manner in the growth of multilayer structures. Ni_3_(2,3,6,7,10,11-hexaiminotriphenylene)_2_, Ni_3_(HITP)_2_, is a conjugated MOF films of which were prepared by interfacial polymerization at the air–water interface. Wu et al. prepared a Ni_3_(HITP)_2_ MOF film and incorporated the resulting film into field-effect transistor (FET) devices, exhibiting p-type semiconductive behaviour, distinguishable on/off ratios, and excellent field-effect hole mobility values as high as 48.6 cm^2^·V^−1^·s^−1^ [223].

A COF film interfacially grown at a liquid–gas interface was also incorporated into a FET device and examined. Feldblyum et al. found an imine-linked COF film growing at the interface of air and a *N*,*N*-dimethylformamide (DMF) solution of COF precursors consisting of benzothiophene and triphenylamine moieties [224]. The interfacially grown film was transferred onto Si substrates in order to fabricate FET devices. The COF-film FET device also exhibited p-type behaviour, an average mobility of 3.0 10^−6^ cm^2^·V^−1^·s^−1^, and an on/off ratio of 850. Imine-linked COF films were also fabricated with the common interfacial polymerization method using LB troughs. Dai et al. newly designed a trisubstituted amine monomer bearing three *n*-hexyl groups [225]. These aliphatic chains are helpful to fix the orientation of the amine monomer when deposited on an air–water interface together with a dialdehyde monomer. The monomers compressed with an LB trough were polymerized with acetic acid as catalyst in the water phase.

Liquid–liquid interfaces are another class of interfaces used for the interfacial polymerization of 2DCPs. Because of the relatively dynamic nature of two liquids, the liquid–liquid interfaces are, in general, less well-defined than liquid-gas interfaces. Hence, the interfacial polymerizations tend to afford thicker films [220]. π‑conjugated nickel bis(dithiolene) complex nanosheets reported by Kambe and co-workers [226] were one of the first representative examples for interfacially polymerized MOF films at the liquid–liquid interface. Two immiscible phases of water and dichloromethane spatially segregate nickel acetate, a metal node precursor, from benzenehexathiol (BHT, a coordinating linker) and confine the MOF formation to the liquid interface. The interfacially grown BHT–Ni network film of 1–2 µm thickness exhibited X-ray diffraction patterns corresponding to a crystalline network structure.

**Figure 11:**
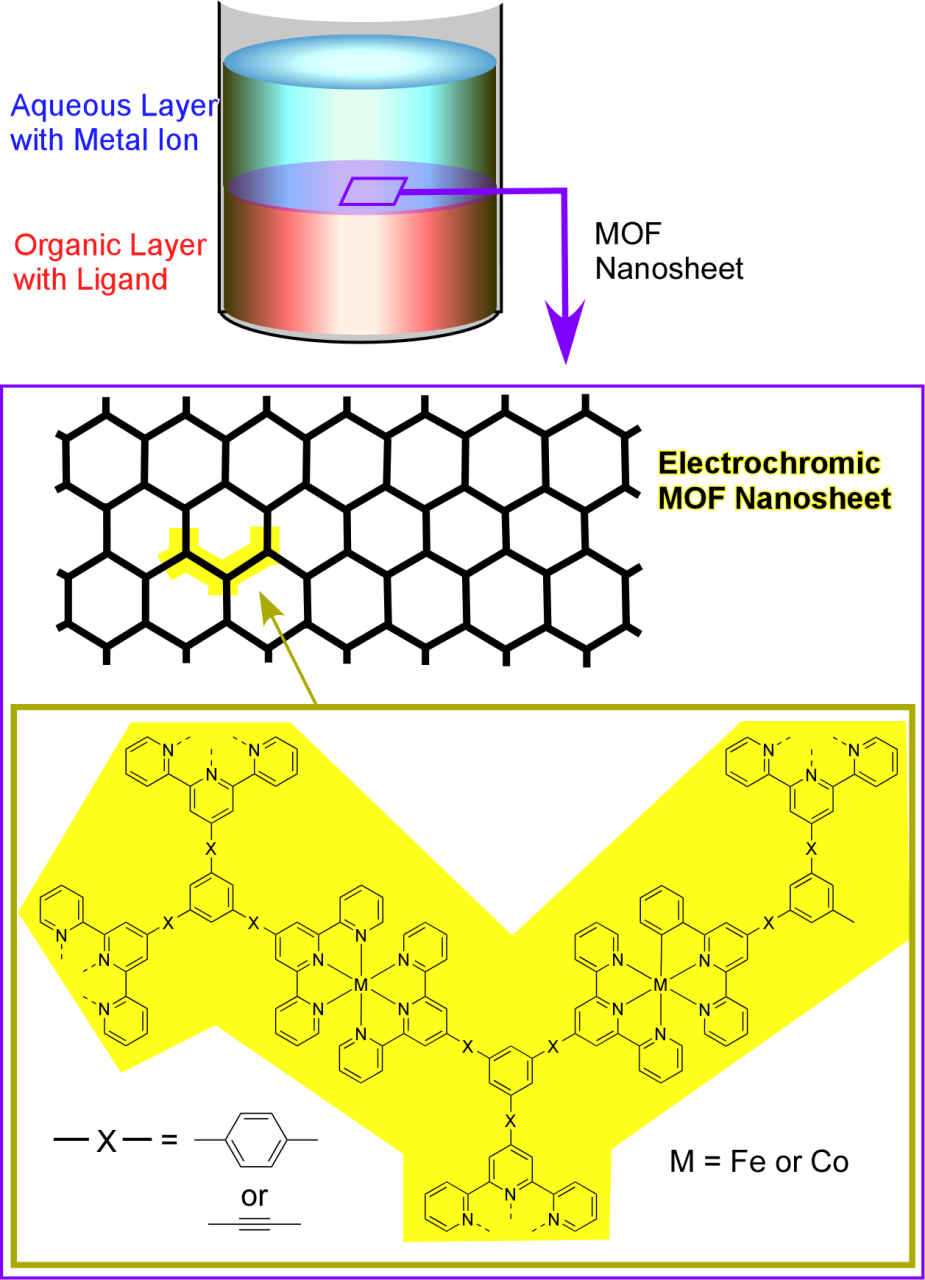
Electrochromic bis(terpyridine)metal complex MOF nanosheets formed through coordination of terpyridine moieties to either cobalt or iron ions. The synthesized films change their colour depending on the oxidation levels of the cobalt and iron ions.

Takada et al. adopted a similar technique to form electrochromic bis(terpyridine)metal complex nanosheets (Figure 11) [227]. The demonstrated network structures are connected by the coordination of terpyridine moieties to either cobalt or iron ions, and the synthesized films change their colour depending on the oxidation levels of the cobalt and iron ions. The colours of those MOF films can be modulated through electrochemical processes.

**Figure 12:**
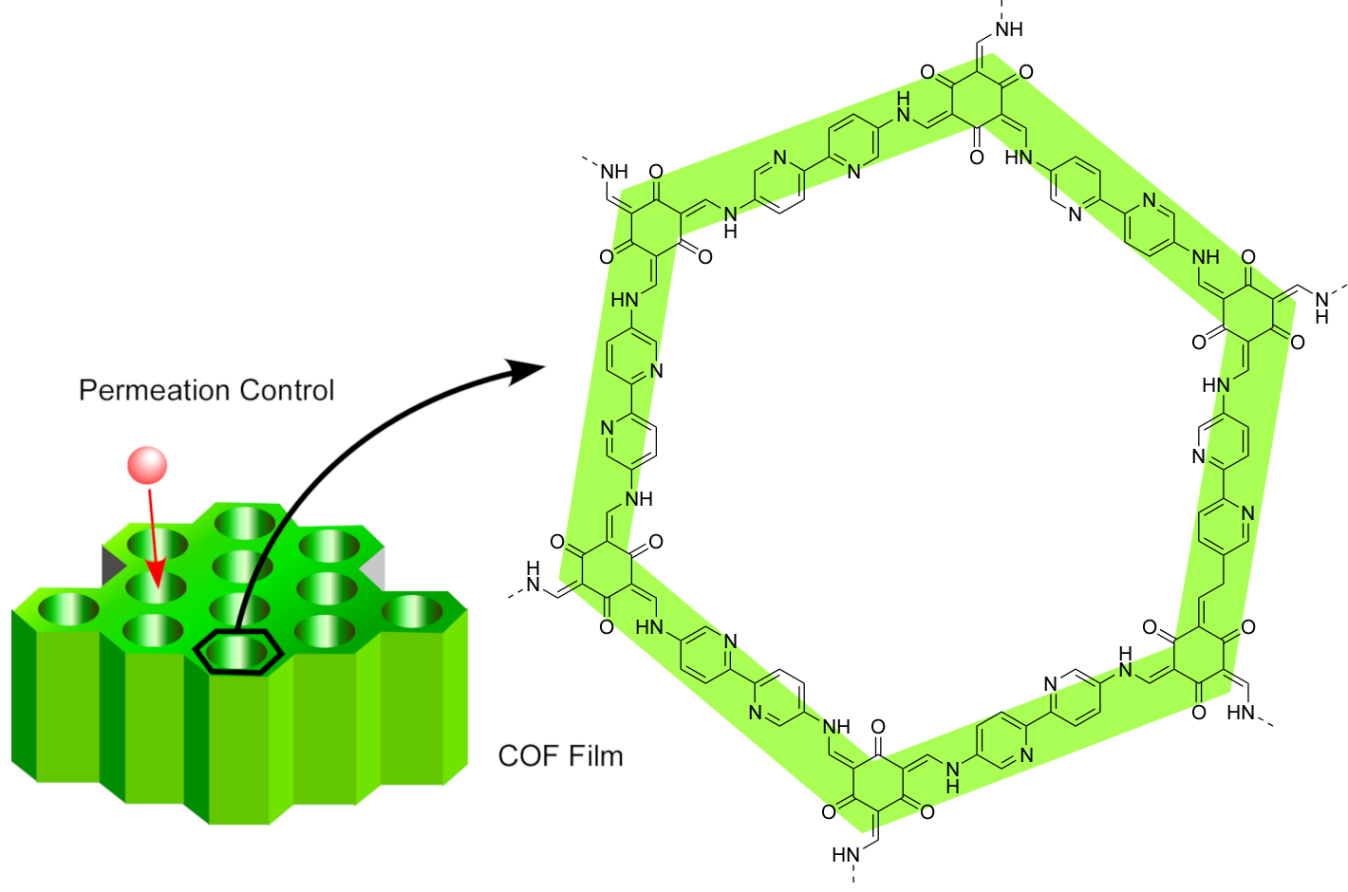
Aqueous solutions of multi-amino-substituted monomers with *p*-toluene sulfonic acid were layered on a dichloromethane solution containing 1,3,5-triformylphloroglucinol to yield large COF films of sub-100 nm thickness.

The interfacial formation of COFs at the liquid–liquid interfaces is more complicated because of the necessity of water-soluble COF precursors. Sahabudeen et al. have overcome this issue by using a hydrophilic dialdehyde monomer, 2,5-dihydroxyterephthalaldehyde (DHTPA) [228]. DHTPA was dissolved in water, and the resulted aqueous solution was layered on top of a chloroform solution of a tetra-substituted amine monomer containing porphyrin. The segregation of the monomers confined the imine formation to the interface and yielded wafer-size multilayer imine-linked COF films. The films grown from the amine monomer containing cobalt porphyrin exhibited catalytic activity for the electrochemical hydrogen generation from water. Dey et al. have dissolved one COF monomer into the aqueous phase by forming amine salts (Figure 12) [229]. Various multi-amino-substituted monomers were treated with *p*-toluene sulfonic acid (PTSA) forming [amine-PTSA] salts, and dissolved into aqueous phases. Each aqueous solution was layered on a dichloromethane solution containing 1,3,5-triformylphloroglucinol (Tp), an aldehyde-derivative COF monomer, yielding large COF films of sub-100 nm thickness. The prepared materials were capable of selective permeation.

**Figure 13:**
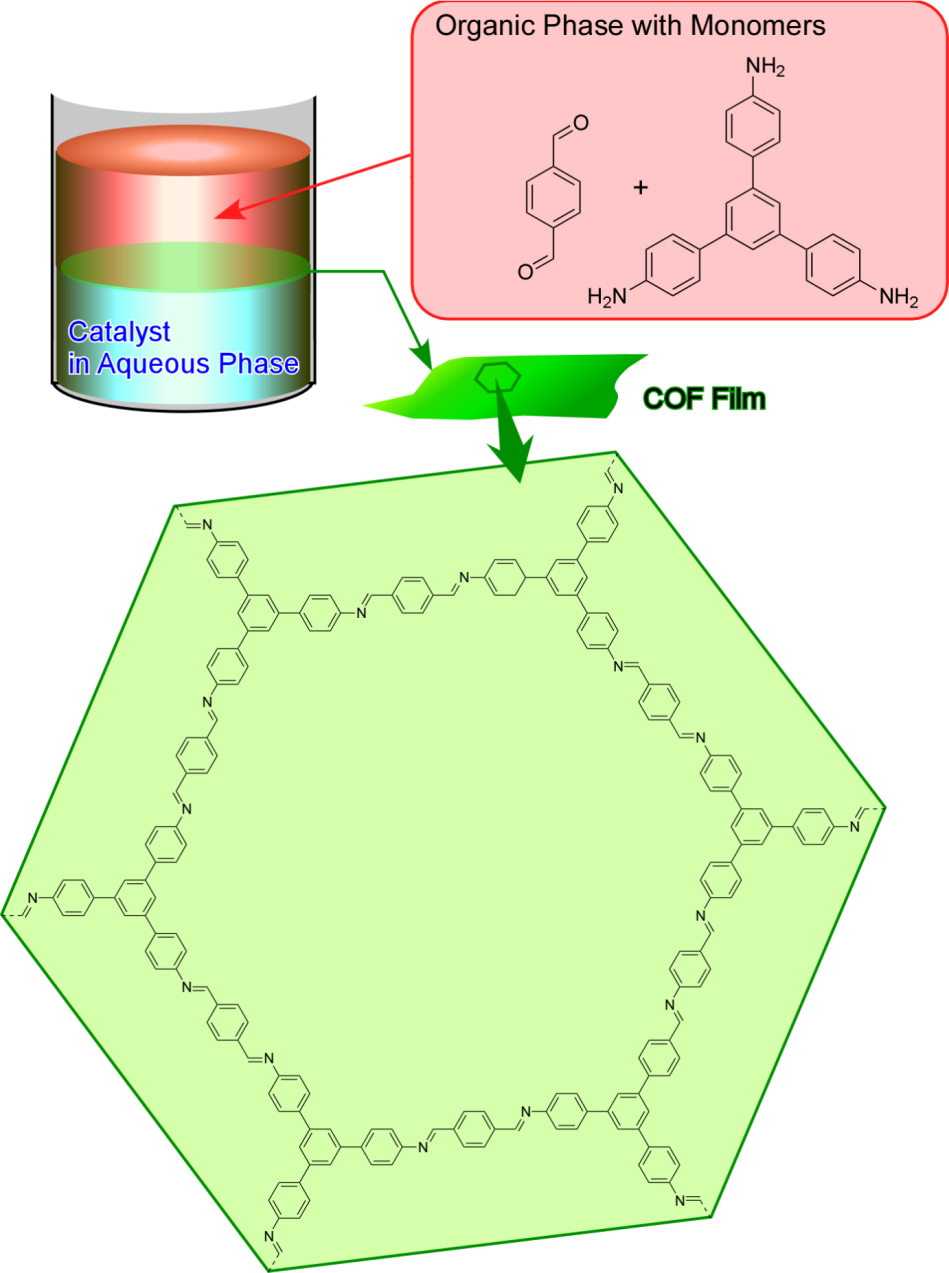
Confined polymerization at the interface by spatially segregating a catalyst from the COF monomers. The formation of imine at the interface of aqueous and organic layer yielded large-area, continuous COF films.

In contrast to the two previous reports in which one of the COF monomers was dissolved into the aqueous phase, Matsumoto et al. confined the polymerization to the interface by segregating the catalyst from the COF monomers (Figure 13) [230]. Scandium triflate, one of the catalysts forming imine-linked COFs [231] in the aqueous phase was isolated from both amine and aldehyde COF monomers dissolved into an organic phase. The two phases in contact with each other induced the COF formation at the interface, forming large-area, continuous COF films (several square centimetres). Depending on the monomer concentrations, the film thickness was tuned from 100 µm to a few nanometres. The COF films made from the methods reported by Dey et al. and Matsumoto et al. were separately examined as separation membranes and exhibited high rejections of water pollutant surrogates from water [229–231]. Considering the high tuning capability of pore-size and functional groups decorating the inner pores of COFs, the separation membranes made out of COFs are promising for water purification technologies including desalination [232].

Interfacial polymerization at liquid interfaces is an already industrialized technique to produce conventional cross-linked polymer films/membranes. Interfacial polymerization of 2DCP films still remains in its infant stage in terms of controlling crystallinity, domain sizes, and generalities. However, the developments on interfacial polymerization of 2DCPs at liquid interfaces will be an important breakthrough for industrializing 2DCP materials.

### 4 Interfacial nanoarchitectonics for nanocarbon materials

#### 4.1 Bottom-up production of nanocarbon materials

Low-dimensional carbon materials, such as carbon nanotubes and graphene derivatives, are now widely used especially in energy and environmental research fields [233]. A lot of attention has also been paid on the creation of novel nanocarbon materials from molecular units and structurally well-defined assemblies from nanocarbon units. Regarding the latter, liquid interfaces often provide important anisotropic fabrication media to synthesize novel types of low-dimensional carbon materials.

**Figure 14:**
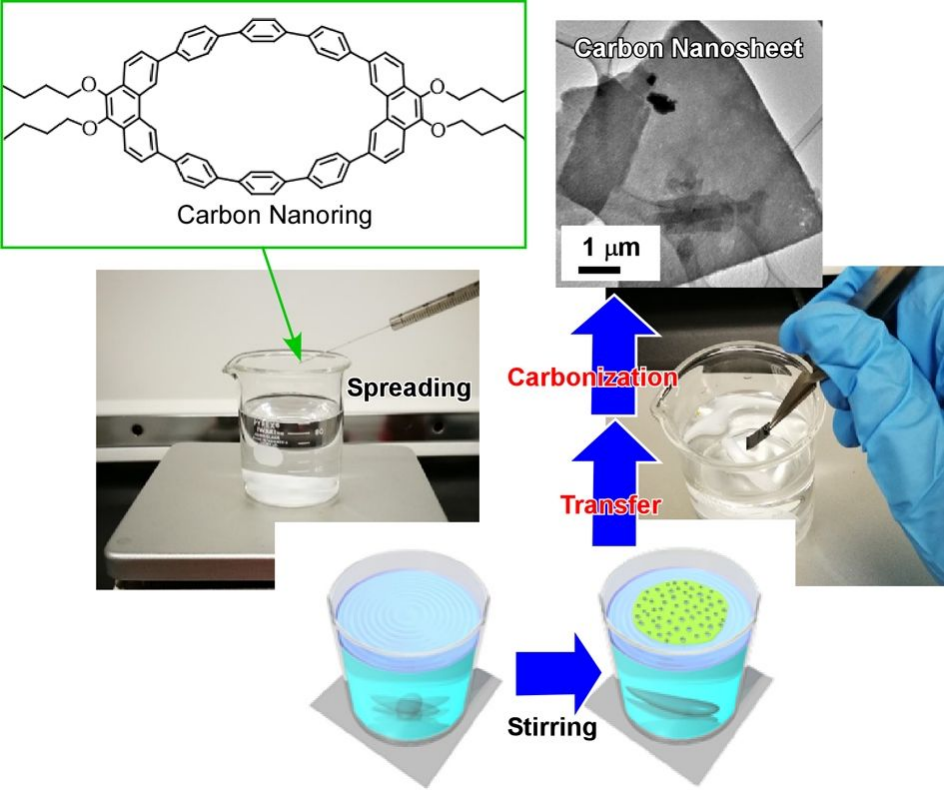
Fabrication of two-dimensional nanocarbon films from a carbon nanoring molecule (9,9′,10,10′-tetrabutoxycyclo[6]paraphenylene[2]-3,6-phenanthrene) via a newly developed vortex LB method at a liquid interface with dynamic flow followed by heat treatment under inert gas atmosphere. Both photographs were reproduced with permission from [68], copyright 2019 The Royal Society of Chemistry; the TEM image was reproduced from [75], copyright The authors.

Recently, Mori et al. successfully demonstrated the fabrication of two-dimensional nanocarbon films from a designed molecular unit, the carbon nanoring molecule (9,9′,10,10′-tetrabutoxycyclo[6]paraphenylene[2]-3,6-phenanthrenylene), by using newly developed vortex LB method at a liquid interface with dynamic flow (Figure 14) [234]. For this bottom-up fabrication, the chloroform solution of the carbon nanoring was dripped at the air–water interface under rotating vortex flow. Appropriate flow rates yielded two-dimensional films of the carbon nanoring molecule with uniform thickness of a few nanometres. The monolayer films were transferred from the water surface to a solid substrate by hand. Further heat treatment under inert gas atmosphere led to the formation of uniform two-dimensional nanocarbon films, so-called carbon nanosheets, with ca. 10 nm thickness with dispersed nanopore structural motifs. The electrical conductivity of the transferred film was significantly increased after the thermal carbonization process. Nitrogen-doping was carried out simply by mixing nitrogen-containing compounds such as pyridine into the original solution of the carbon nanoring molecule. The prepared nitrogen-doped carbon nanosheets exhibited a higher electrical conductivity than the non-doped ones did. It should be noted that these nanomaterial fabrications can be conducted by using solely very common apparatuses such as beaker, stirrer, and tweezers. Therefore, the proposed method can be more generalized and extended even to industrial applications.

**Figure 15:**
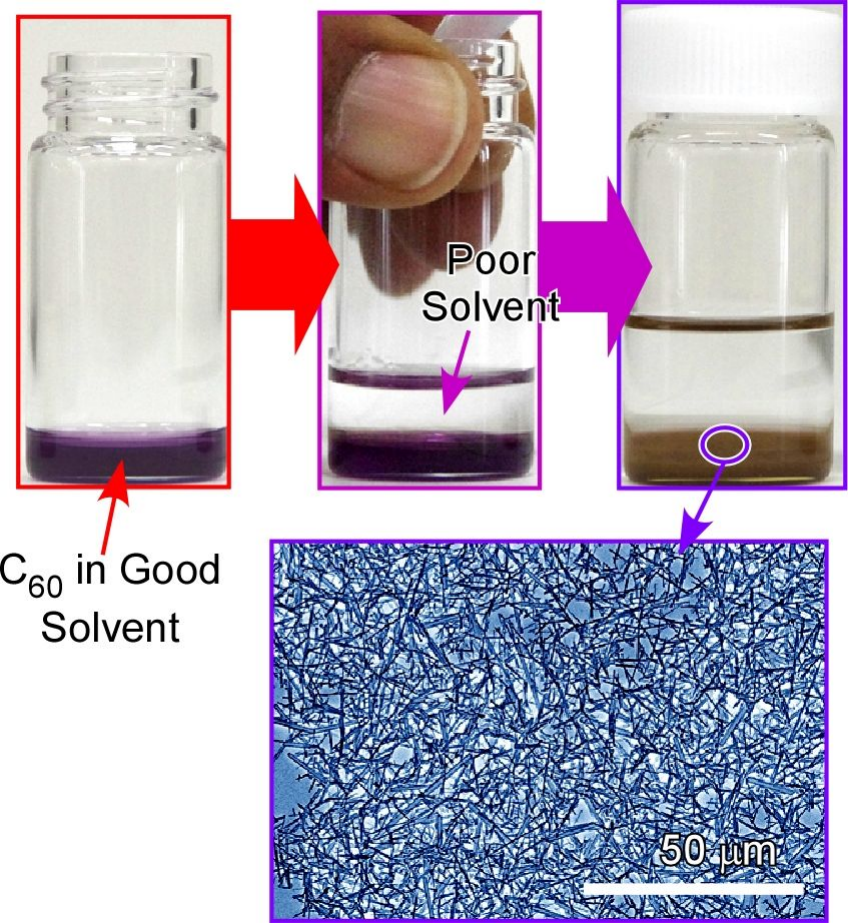
Liquid–liquid interfacial precipitation to fabricate fullerene assemblies (crystals) with various morphologies (in this case, assembly of one-dimensional nanowhiskers). Fullerene molecules, such as C_60_ and C_70_, are dissolved in a liquid phase (good solvent) that contacts an immiscible liquid phase in which the molecules are poorly soluble (poor solvent).

For the bottom-up fabrication of nanocarbon materials through self-assembly processes, the use of fullerene (especially, C_60_) molecules as assembling components are both technically and scientifically attractive [235]. In addition to the technical importance of fullerene in many applications including physical devices and biomedical usages, assembly processes from completely symmetrical zero-dimensional objects with single elemental composition (carbon) are an intriguing fundamental topic in supramolecular chemistry [236]. Miyazawa and co-workers initiated a simple but highly useful method, i.e., liquid–liquid interfacial precipitation, to fabricate fullerene assemblies (crystals) with various morphologies (Figure 15) [237–239]. Fullerene molecules, such as C_60_ and C_70_, are dissolved in a liquid phase (good solvent) that contacts an immiscible liquid phase in which the molecules are poorly soluble (poor solvent). The formation of crystalline assemblies of fullerene molecules is induced by supersaturation at the liquid–liquid interface. For example, C_60_ rods or needles can be obtained at the interface between a saturated solution of C_60_ in toluene and isopropyl alcohol as poor solvent.

#### 4.2 One-dimensional fullerene assembly materials

Shrestha and co-workers have extended the research on dimensionally controlled assemblies of fullerene molecules through liquid–liquid interfacial precipitation [240]. For example, the conversion from one-dimensional structures to three-dimensional morphologies of C_60_ rods and tubes was carried out via a surfactant-assisted process in liquid–liquid interfacial precipitation [241]. At the interface between butanol and benzene, C_60_ typically assembles into one-dimensional superstructures (rods and tubes). However, by adding surfactants to those interfacial systems, the morphology of the assemblies can be altered to three-dimensional objects. The final morphology highly depends on type and concentration of the surfactants. When the non-ionic surfactant diglycerol monolaurate was added to butanol (0.01%), flower-like three-dimensional objects were precipitated at the interface with benzene. Detailed morphological analyses with electron microscopy techniques revealed that the surfactants did not basically alter primarily the one-dimensional structures of the formed assemblies. Instead, they seemed to promote super-lattice formation constructing three-dimensional flowers form the same one-dimensional rods observed in non-surfactant systems (tubes).

**Figure 16:**
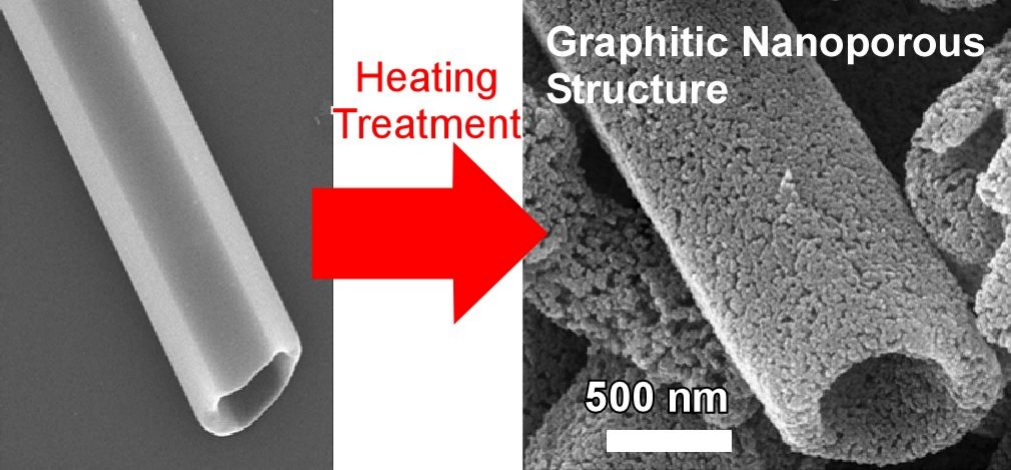
Conversion of one-dimensional C_60_ nanotubes precipitated at liquid-liquid interface to one-dimensional carbon materials having sp^2^-carbon-based π-electron-rich robust mesoporous frameworks through carbonization at 2000 ^o^C in vacuum.

The thermal conversion of one-dimensional fullerene crystalline assemblies at extremely high temperatures resulted in highly graphitic one-dimensional carbon materials as demonstrated by Shrestha and co-workers [242]. One-dimensional C_60_ nanorods and nanotubes precipitated at liquid–liquid interface were fully carbonized at 2000 °C in vacuum, resulting in morphology-preserved one-dimensional carbon materials with sp^2^-hybridised π-electron-rich robust frameworks (Figure 16). Due to their highly aromatic nature, microbalance sensors with the synthesized one-dimensional carbon materials on a quartz crystal microbalance plate exhibited superior sensing properties for aromatic toxic gasses. In addition, these graphitic carbon materials exhibit excellent electrochemical capacitance, suggesting possible usages in electrochemical and electrical applications. A similar nanoarchitectonics strategy was adopted to C_70_ molecules. The one-dimensional carbon materials prepared through high-temperature carbonization of C_70_ crystalline assemblies showed high specific capacitances at a high current density and scan rate [243]. These nano-engineered one-dimensional carbon materials might be useful as electrode materials for supercapacitors.

Shrestha, Acharya, and co-workers investigated the optoelectronic properties of one-dimensional C_60_ nanorods prepared in ultra-rapid (5 s) processes of liquid–liquid interfacial precipitation at room temperature [244]. Dominant excitonic charge transfer transitions within the nanorods was confirmed by steady-state optical spectroscopy. Photovoltaic cells with one-dimensional C_60_ nanorods as active layer sandwiched by an indium tin oxide anode and an aluminium cathode exhibited enhanced photovoltaic capabilities. It also led to a significant enhancement of photogenerated charge carriers as compared to similar cells prepared with pristine C_60_ molecules. C_60_ molecules in a one-dimensional van der Waals solid preserve the electronic structure of C_60_, but they crystallise in a hexagonal close-packed structure that is different from the cubic crystal structure of pristine C_60_ molecular crystals. This fact suggests that crystal lattice and molecular packing within low-dimensional fullerene assemblies significantly modify the optoelectronic properties. The rapid synthesis with the possibility to scale-up and the enhanced optoelectronic properties make the above-mentioned nanoarchitectonics strategy for one-dimensional fullerene nanorods a promising approach for applications in photosensitive devices.

Ji, Shrestha, and co-workers investigated the effects of the intercalation of polycyclic aromatic compounds, such as naphthalene, anthracene, and pyrene, on the formation of one-dimensional C_60_ nanowhiskers in liquid–liquid interfacial precipitation processes [245]. The intercalation of polycyclic aromatic compounds generally modifies the growth of fullerene one-dimensional crystals depending on intercalation species. While anthracene and pyrene led to an increased porosity of the structures, the structural characteristics of those without intercalator compounds were preserved in the presence of naphthalene. In addition, intercalation of the polycyclic molecules significantly modified the spectral emissions of the fullerene assemblies probably due to effects of molecular packing on the electron transfer within the assembled structures.

Acharya, Shrestha, and co-workers decorated one-dimensional C_60_ nanorods with zero-dimensional Ag nanoparticles that were used as substrates for surface-enhanced Raman scattering (SERS) to detect model targets such as rhodamine 6G with high sensitivity [246]. This system provides dispersed SERS substrates that can be evaluated by confocal Raman imaging. The nanoarchitectonic materials work as freestanding efficient plasmonic substrates for molecular detection.

**Figure 17:**
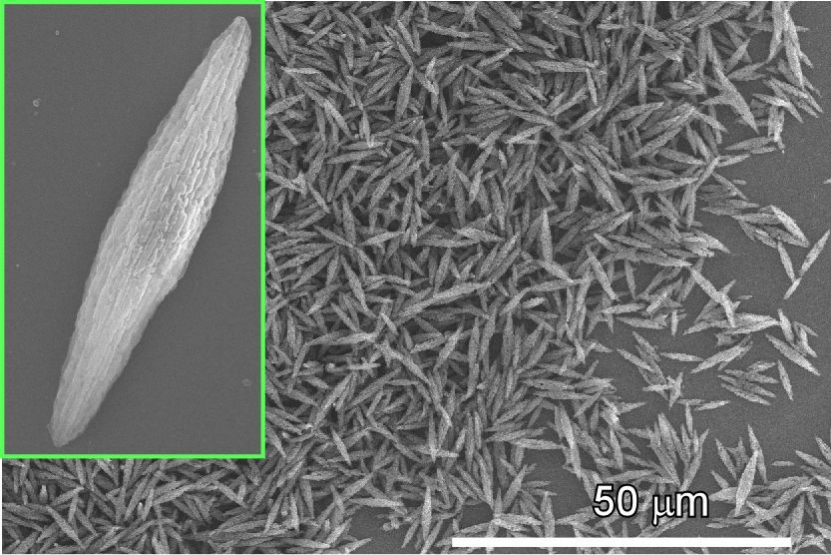
Nanoporous bitter-melon-shaped C_60_ crystals with face-centered cubic lattice fabricated through liquid-liquid interfacial precipitation methods between isopropyl alcohol and C_60_ solution in dodecylbenzene.

Nanoporous bitter-melon-shaped C_60_ crystals with face-centred cubic lattice were fabricated through liquid–liquid interfacial precipitation from 2-propanol and C_60_ solution in dodecylbenzene as reported by Shrestha and co-workers (Figure 17) [247]. Quartz crystal microbalance sensors coated with the bitter-melon-shaped objects exhibited excellent sensing properties for aromatic vapours with sensitivities in the order of aniline > toluene > benzene > ethanol > hexane > cyclohexane > methanol > water. The obtained nanoporous low-dimensional C_60_ assemblies provide advantageous features of easy diffusion and promoted π–π interactions for facile sensing.

Saran and Curry reported the use of one-dimensional C_60_ crystals prepared through liquid–liquid interfacial precipitation between *m*-xylene and isopropyl alcohol for visible-spectrum photodetectors [248]. Additional materials or two metal contacts are not necessary in the fabricated photodetectors. These devices made solely from carbon can be used as an alternative to commercial photodetector devices with CdS and CdSe. Enhanced photoluminescence and photoelectrochemical properties of one-dimensional Lu_2_@C_82_ nanorods prepared through liquid–liquid interfacial precipitation between carbon disulfide and 2-propanol were demonstrated by Lu and co-workers [249]. Photoluminescence of the one-dimensional Lu_2_@C_82_ nanorods was remarkably enhanced compared to the pristine Lu_2_@C_82_ powder. The increased charge carrier transport would be also useful for applications with photoelectric purposes such as photodetectors.

#### 4.3 Two- and three-dimensional, and hierarchic fullerene assembly materials

**Figure 18:**
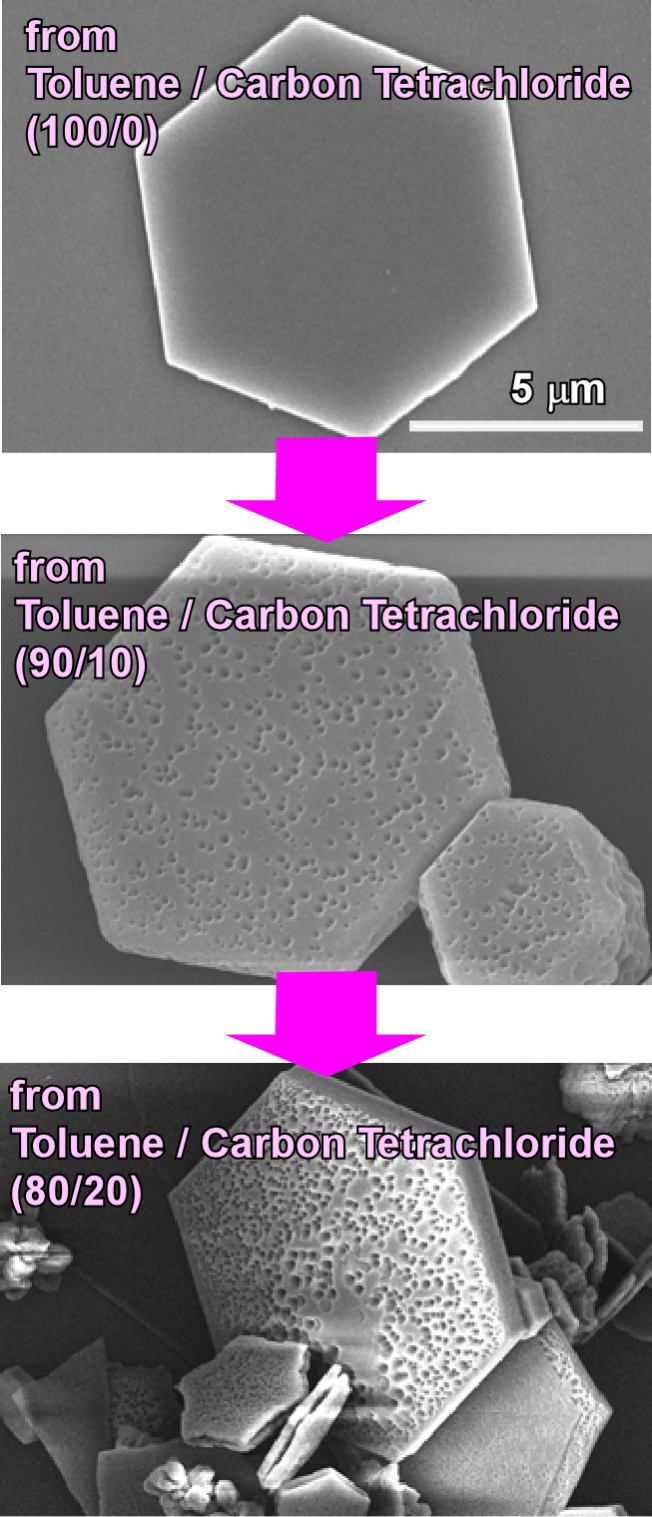
Two-dimensional C_60_ hexagonal nanosheets with hierarchic pore systems of macropores and mesopores prepared by liquid–liquid interfacial precipitation from isopropyl alcohol and mixtures of benzene and carbon tetrachloride. The porosity can be tuned through the mixing ratio between benzene and carbon tetrachloride. SEM images were reproduced with permission from [68], copyright 2019 The Royal Society of Chemistry.

One of the biggest advantages of liquid–liquid interfacial precipitation is the capability of creating nanomaterials with different dimensionalities just by changing the combination of liquids to form the interface. For example, Shrestha and co-workers successfully prepared two-dimensional C_60_ hexagonal nanosheets with hierarchic pore structures of macropores and mesopores just by changing the solvent combination (Figure 18) [250]. Liquid–liquid interfacial precipitation processes with isopropyl alcohol/benzene and isopropyl alcohol/carbon tetrachloride provided one-dimensional rods and two-dimensional hexagon nanosheets, respectively. While these objects do not possess porous interior structures, the use of good solvents (benzene and carbon tetrachloride) with isopropyl alcohol as poor solvent yielded nanosheets with pores depending on the mixing ratio between benzene and carbon tetrachloride. Incorporation of 30% carbon tetrachloride changes the morphology from one-dimensional rods to two-dimensional hexagonal sheets by preventing the sheets from rolling up to rods. Carbon tetrachloride contents of more than 50% yielded porous structures of two-dimensional hexagon nanosheets. At 90% carbon tetrachloride content, the average pore size became ca. 400 nm. These integrated two-dimensional structures would be nanoarchitectonics pieces for the fabrication of sensitive sensors, organic solar cells, and miniaturized organic superconductors.

**Figure 19:**
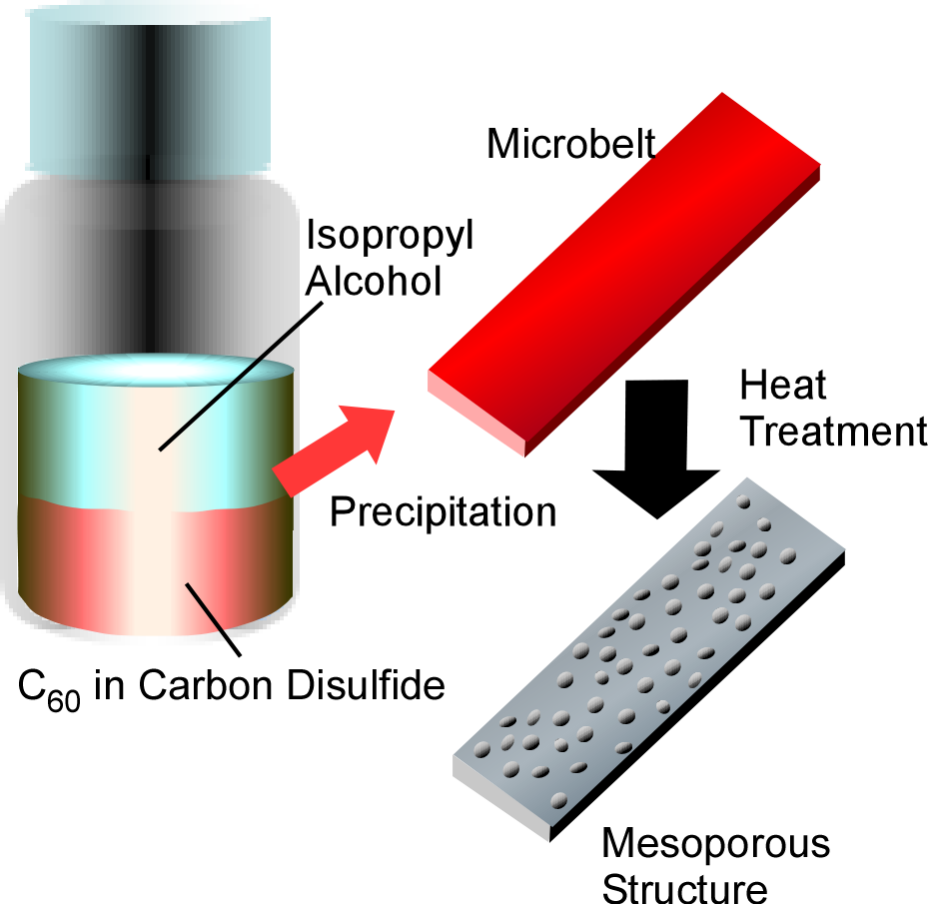
Synthesis of two-dimensional mesoporous C_60_-based carbon microbelts by liquid–liquid interfacial precipitation using a carbon disulfide solution of C_60_ and isopropyl alcohol followed by high-temperature heat treatment.

As a functional development of two-dimensional fullerene objects, Ji, Shrestha, and co-workers reported the synthesis of two-dimensional mesoporous carbon microbelts and demonstrated their usage as electrode material for electrochemical supercapacitors (Figure 19) [251]. Two-dimensional belt-like mesoporous structures can be fabricated from C_60_ molecules by liquid–liquid interfacial precipitation using a carbon disulfide solution of C_60_ and isopropyl alcohol. Under optimized conditions, these mesoporous C_60_ microbelts extended to lengths of the order of centimetres. Heat treatment of the obtained C_60_ microbelts converts them into two-dimensional amorphous carbon microbelts at 900 °C and their dense graphitic versions at 2000 °C. Especially the former carbon material exhibited excellent electrochemical supercapacitive performance due to the enhanced surface area and the robust mesoporous framework motifs. The hierarchical bimodal pore nature throughout the carbonaceous frameworks results in efficient charge storage and rapid ion transport. Superior cycling stability without any capacity losses even after 10000 charge/discharge cycles was also confirmed.

**Figure 20:**
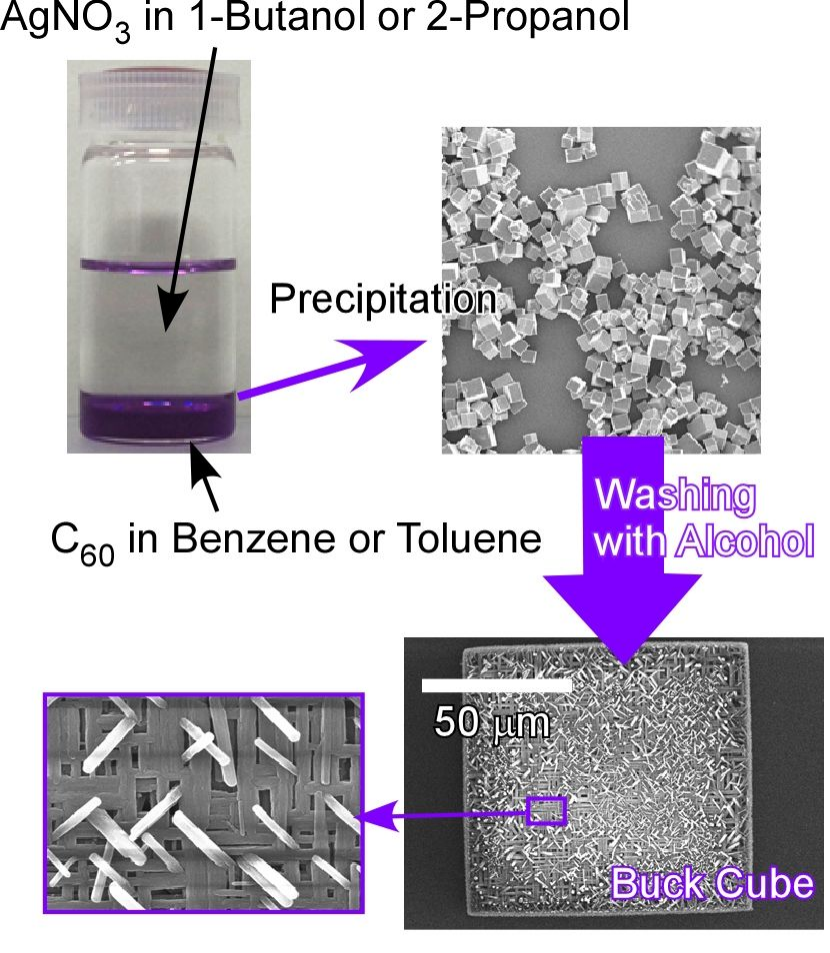
Highly integrated three-dimensional Bucky cubes synthesized by liquid–liquid interfacial precipitation of Olmstead's crystalline C_60_–Ag(I) organometallic heteronanostructure and the subsequent structural transformation upon post-solvent treatment.

Furthermore, preparations of three-dimensional and hierarchic structures of fullerene assemblies through liquid–liquid interfacial precipitation have been successfully reported. Shrestha et al. reported the synthesis of highly integrated three-dimensional Bucky cubes through liquid–liquid interfacial precipitation and structural transformation by post-solvent treatment (Figure 20) [252]. Three-dimensional cubic structures were precipitated as Olmstead’s crystalline C_60_–Ag(I) organometallic hetero-nanostructures [C_60_{AgNO_3_}_5_] at the interface between a saturated benzene solution of C_60_ and an ethanol solution of silver(I) nitrate. The formed cubic structures underwent structural transformation upon exposing them to aliphatic alcohols of low molecular weight. The transformation of smooth-faced crystals to interpenetrated networks of one-dimensional needle crystals occurred while preserving the cubic shape. Several potential applications based on their electronic and optical properties can be expected for the obtained highly integrated fullerene assemblies.

**Figure 21:**
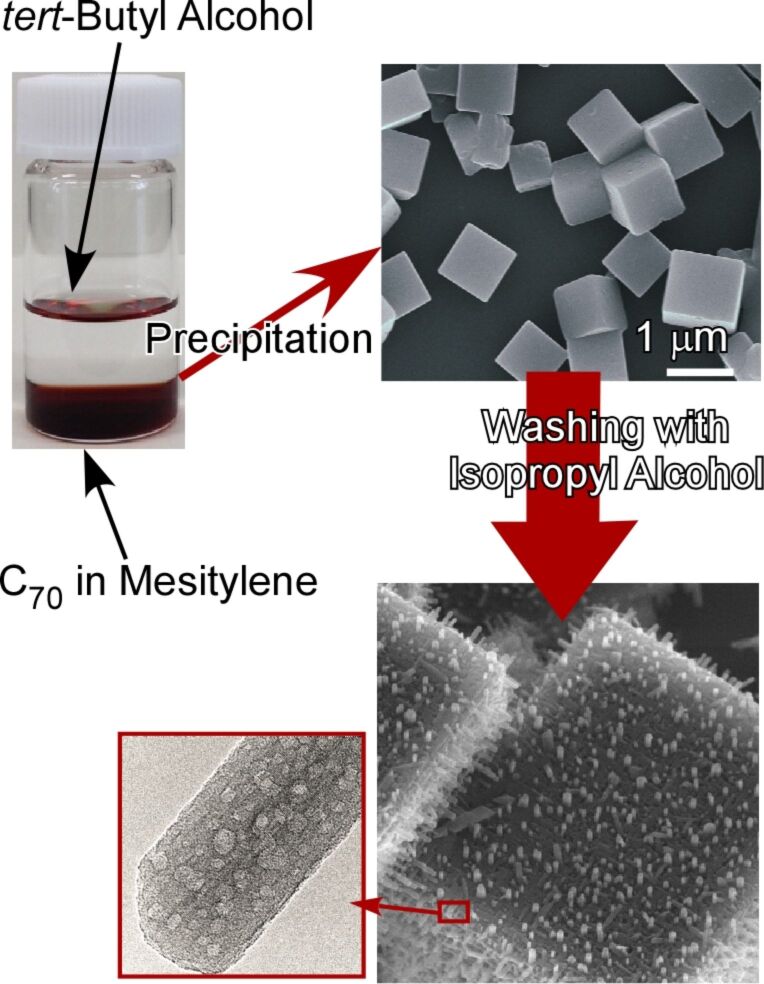
Three-dimensional cubic structures can be fabricated from C_70_ molecules through an ultrasound-assisted liquid–liquid interfacial precipitation from *tert*-butyl alcohol and mesitylene. The formed cubes can be further transformed into needle-on-cube (one-dimensional structure on the surface of three-dimensional object) structures simply by washing with isopropyl alcohol at room temperature. Reproduced from [75], copyright 2019 The authors.

As reported by Shrestha and co-workers, three-dimensional cubic structures can be fabricated from C_70_ molecules through an ultrasound-assisted liquid–liquid interfacial precipitation from *tert*-butyl alcohol and mesitylene [253]. In this modified method, mild sonication was applied for a short period of time after appropriate incubation time. The resulting C_70_ cubic objects were further transformed into needle-on-cube (one-dimensional structure on the surface of three-dimensional object) structures simply by washing with isopropyl alcohol at room temperature (Figure 21). The growth directions and diameters of the nanorod-like C_70_ one-dimensional structures can be tuned through the washing conditions. Interestingly, the formed nanorod structures possess mesoporous features, which makes the entire structure fully hierarchic. Quartz crystal microbalance sensors modified with these hierarchic C_70_ assemblies exhibited an excellent sensitivity to aromatic molecules in their vapour phase probably due to facile diffusion through the porous structure, high surface-area contact and advantageous π–π interaction. The formation of low-dimensional objects from a flat surface efficiently increases the surface area. This strategy of converting low-dimensional structures is beneficial for certain application such as sensing and drug delivery where contact of materials to external media is crucial.

**Figure 22:**
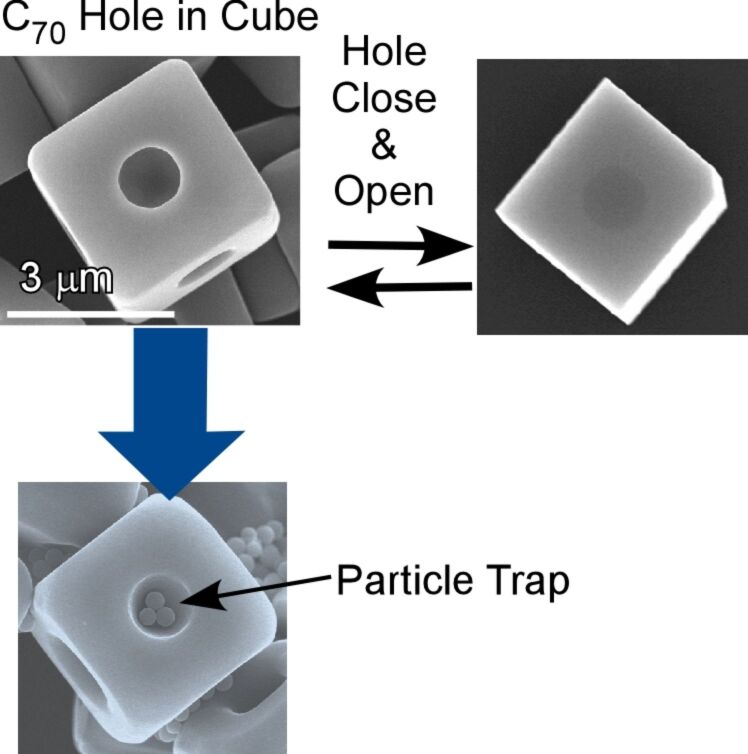
Hole-in-cube structures made from C_70_ molecules with hole closing and re-opening and particle-trap capability. Reproduced from [75], copyright 2019 The authors.

Shrestha and co-workers demonstrated the manipulation of microscopic hole structures on the surface of cubic assemblies of C_70_ molecules leading to hole-in-cube structures (Figure 22) [254]. Open-hole cubes, in which microscopic holes are formed at center of every face, were fabricated through dynamic liquid–liquid interfacial precipitation from *tert*-butyl alcohol and mesitylene. In the dynamic procedure, a mesitylene solution of C_70_ molecules was rapidly added into *tert*-butyl alcohol and the resulting mixture was further incubated. The closing and re-opening of holes can be controlled through addition of excess C_70_ molecules and local electron beam irradiation, respectively. Interestingly, the fabricated holes have the capability to discriminate macro-size particles. The holes selectively accommodate graphitic carbon particles instead of resorcinol–formaldehyde resin particles of similar shape and size. Favourable π–π interactions at the sp^2^-rich interior surface of the open holes are responsible for this selective capture of microscopic particles.

The preparation of mesoporous crystalline cubes of C_70_ molecules with unusually enhanced photoluminescence has been recently reported by Shrestha and co-workers [255]. The mesoporous C_70_ cubes can be prepared by applying a mild heat treatment process to the liquid–liquid interfacial precipitation from *tert*-butyl alcohol and mesitylene. Initially formed crystalline C_70_ cubes were transformed into mesoporous C_70_ cubes via intermediate one-dimensional structures. The resulting mesoporous C_70_ cubes showed enhanced photoluminescence probably due to their highly crystalline framework structures. In addition, these materials exhibited superior electrochemical supercapacitance over pristine C_70_ because of their increased electrochemically active surface areas.

Shrestha et al. also reported a modified method of liquid–liquid interfacial precipitation for fullerene nanoarchitectonics using non-ionic surfactants such as diglycerol monolaurate and diglycerol monomyristate [256]. The liquid–liquid precipitation from isopropyl alcohol and ethylbenzene usually provided one-dimensional structures. These were, however, transformed into Konpeito candy-like three-dimensional crystalline structures in the presence of the above surfactants in the ethylbenzene phase. Furthermore, the fabricated three-dimensional objects can be graphitized by heat treatment at 2000 °C. The obtained carbon materials show a high potential for applications in energy storage supercapacitor devices.

Liquid–liquid interfacial precipitation methods with the strategies of mixing components and conjugating molecules also create interesting results. Lu, Guldi, and co-workers investigated the co-crystallization of C_70_ and (metallo)porphyrins through liquid–liquid interfacial precipitation to give two-dimensional nanosheet structures [257]. Single crystal X-ray diffraction studies on the fabricated objects confirmed equimolar fractions of these two components. As indicated by steady-state absorption spectroscopy and fluorescence spectroscopy, a strong charge transfer interaction resulted in the charge separation with one-electron reduced C_70_ and one-electron oxidized (metallo)porphyrins. Li and co-workers reported the formation of superstructures of a C_60_–adamantane conjugate through liquid–liquid interfacial precipitation with chloroform as the good solvent [258]. Diverse morphological structures with various dimensionalities such as spheres, fibers, plates, nanoflowers, cubes and microparticles were obtained.

**Figure 23:**
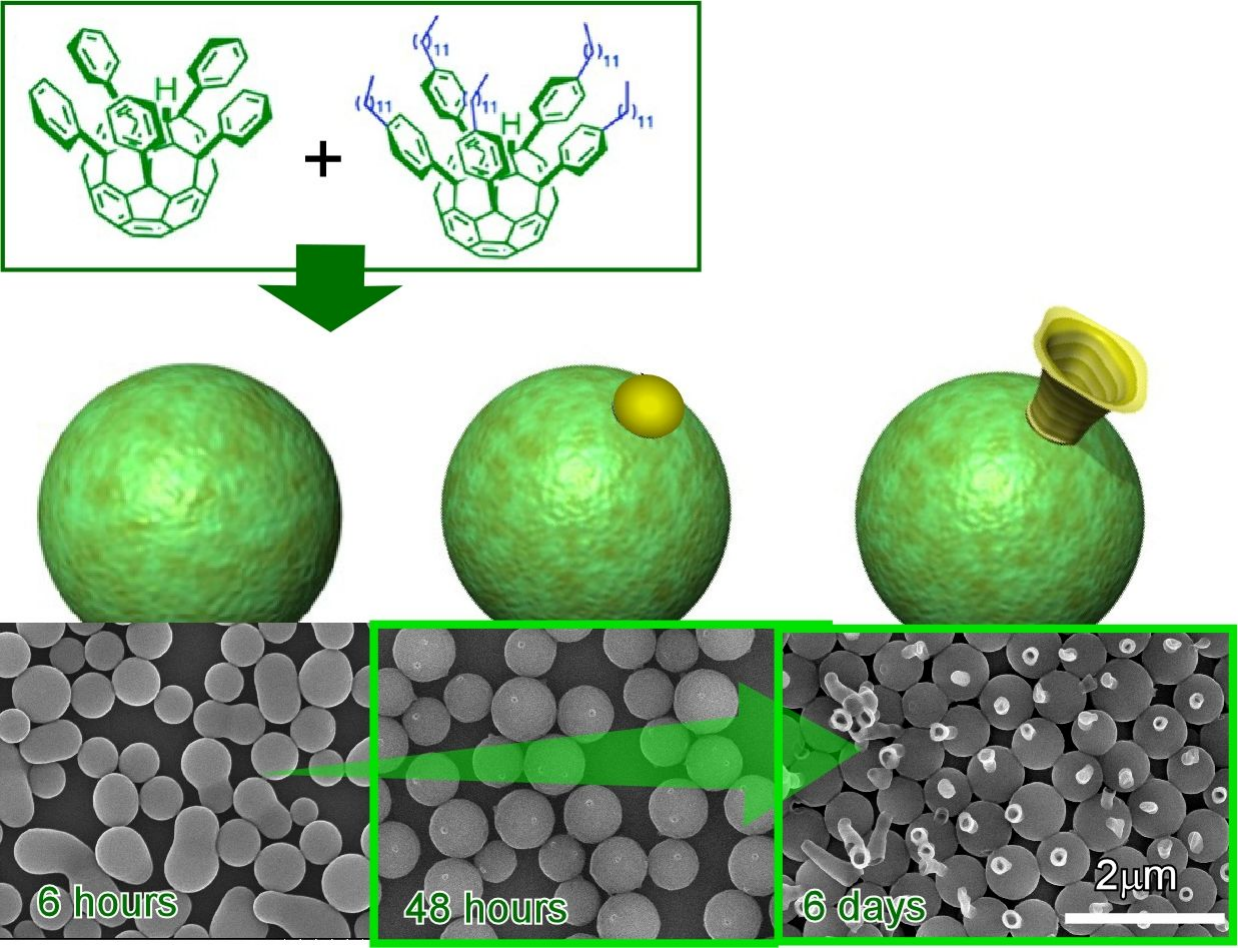
Time-dependent shape shift of co-assemblies of two fullerene derivatives, pentakis(phenyl)fullerene and pentakis(4-dodecylphenyl)fullerene from egg-like structures to tadpole-like structure regarded as supramolecular differentiation. Reproduced from [75], copyright 2019 The Authors.

As an interesting example of two-component fullerene assemblies at liquid–liquid interfaces, Minami, Shrestha, and co-workers demonstrated time-dependent shape shifts of co-assemblies of two fullerene derivatives, pentakis(phenyl)fullerene and pentakis(4-dodecylphenyl)fullerene (Figure 23) [259]. Structural shifts from egg-like structures to tadpole-like structures are regarded as supramolecular differentiation. At the interface of isopropyl alcohol and toluene, egg-like structures were first formed from the mixture of these two components. One or multiple domains of pentakis(4-dodecylphenyl)fullerene appeared on the spherical assemblies of pentakis(phenyl)fullerene using appropriate mixing ratios and appropriate incubation times. From the phase-separated domains, one-dimensional tubular structures of pentakis(4-dodecylphenyl)fullerene preferentially growth as tails upon gentle sonication. The observed supramolecular differentiation can be regarded as the materials-science-based analogue of embryonic development.

### 5 Interfacial nanoarchitectonics for biomaterials

Well-designed biomolecular units should be powerful tools for nanoarchitectonics of low-dimensional materials [260–262]. Asymmetric motifs of biomolecular units can be well designed in many cases and the synthesized asymmetric units are often assembled into low-dimensional structures. As summarized in a recent review article by Matsuura [263], assembled structures of various shapes can be nanoengineered from well-designed biomolecular units such as carbohydrate-conjugated oligodeoxyribonucleotides and three-way junctions of DNA assemblies and oligopeptides. Uses of the assembled low-dimensional materials for drug carriers, ligand-displaying scaffolds, and platform for platforms are anticipated. Sawada and Serizawa explained in their recent review the use of M13 phages for asymmetrical assemblies [264]. Although M13 phages are generally known as useful scaffold for phage-display technology, they utilized the M13 phages as one-dimensional building block for novel liquid crystalline materials. These unique approaches can open a new avenue for phage-based soft materials. He and Xu reported a novel concept, namely, instructed assembly (iAssembly) [265], which can yield ordered low-dimensional assemblies as the consequence of at least one trigger event. The instructed assembly can be well related to molecular processes to control cell fate.

These examples strikingly indicate that biomaterials have a high potential as building blocks for nanoarchitectonics of functional low-dimensional structures. Restricting these assembly processes two low-dimensional media with certain degrees of motional freedom and diffusional restriction would result in functional low-dimensional materials with attractive biological functions. Liquid interfaces (especially aqueous interfaces) would be appropriate media for this purpose. However, the high surface tension at the air–water interface is disadvantageous for protein assembly because undesirable transformations of secondary structures might be induced by the high surface tension. One method to suppress surface denaturation during two-dimensional protein assembly was proposed by Fromherz. The adsorption of proteins from an underneath aqueous subphase to a lipid monolayer at the air–water interface in a multi-compartment trough can prevent undesirable denaturation of proteins [266–267].

**Figure 24:**
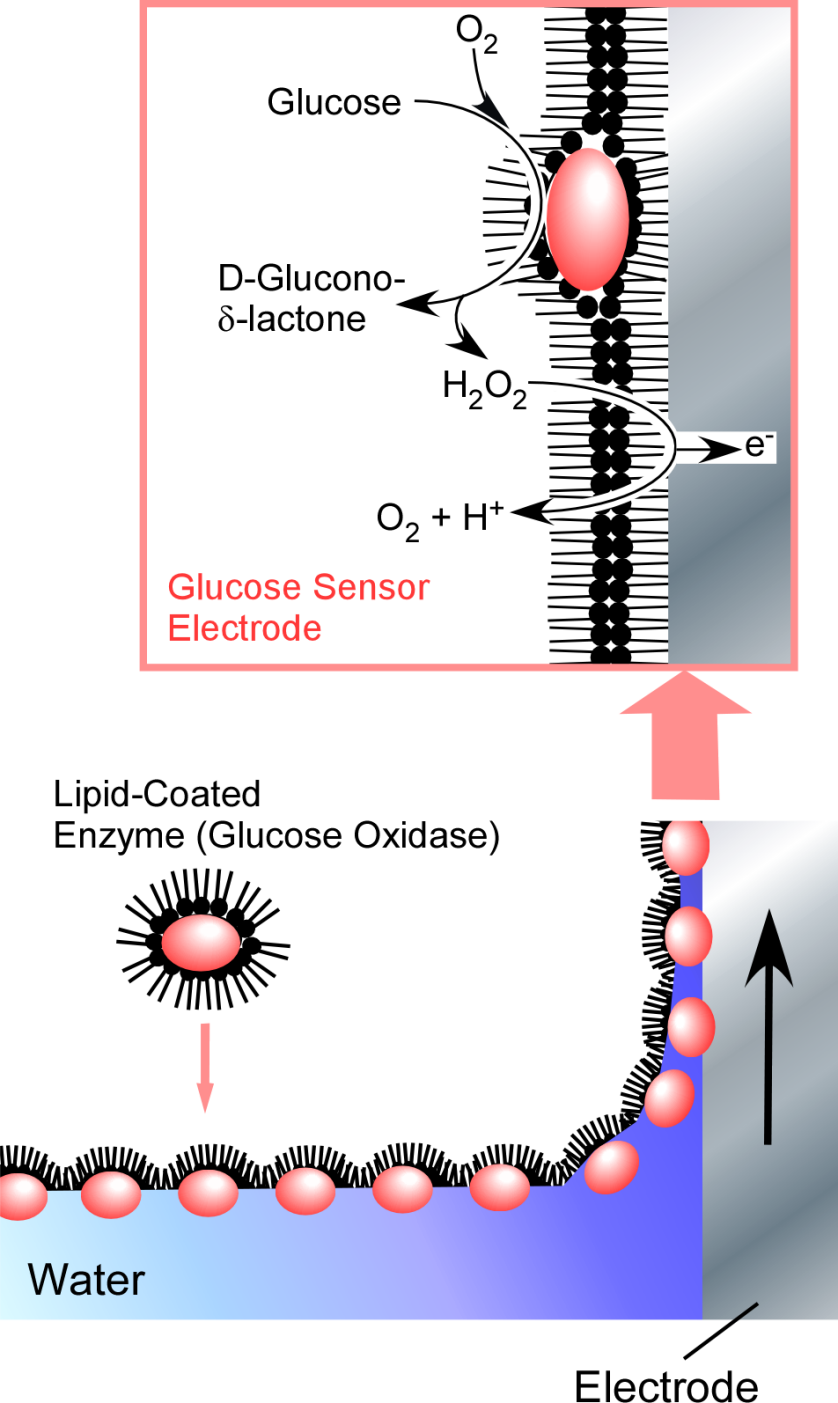
A monolayer of glucose oxidase complexed with a cationic lipid was transferred onto a surface of an electrode for glucose detection sensors.

Because the method by Fromherz may waste unused proteins in the subphase, it is not always suitable for the two-dimensional assembly of precious biomaterials. In order to overcome this drawback, Okahata and co-workers used lipid-coated proteins for a LB process at the air–water interface [268–269]. Water-soluble biomolecules such as proteins were mixed with aqueous vesicles of appropriate lipid molecules, resulting in a water-insoluble lipid–biomolecule complex. For example, glucose oxidase was complexed with a cationic lipid to provide water-insoluble materials that were soluble in organic solvents. A benzene solution of the resulting complex was dripped at the air–water interface to give a monolayer film of glucose oxidase and the lipid. The, only the amount of glucose oxidase needed for monolayer formation is required and denaturation of glucose oxidase is avoided by lowering the surface tension through the presence of the lipid. The monolayers were transferred onto a surface of electrodes, leading to glucose detection sensors (Figure 24). Similarly, the presence of additional components such as lipids and polymers is advantageous for other methods of fabricating two-dimensional layered films, such as layer-by-layer assembly [270–273]. Single-enzyme and multi-enzyme reactors were successfully demonstrated [274–276].

**Figure 25:**
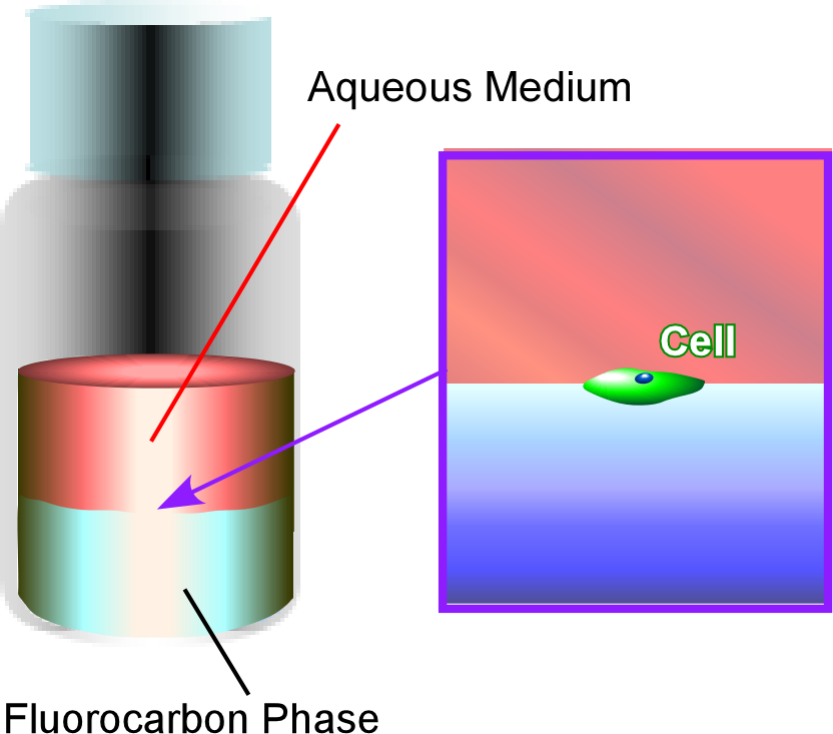
Cultures, organizations, and differentiation controls of living cells a liquid-liquid interface between aqueous media and fluorocarbon phase.

In addition to the above-mentioned basic efforts in the nanoarchitectonics of bio-components through processes at liquid interfaces, there are more advanced strategies currently under research. For example, the culture, the organization, and the control over the differentiation of living cells have been investigated recently at liquid interfaces. Minami et al. pioneered in the cell differentiation control at a liquid–liquid interface (Figure 25) [277]. Several other research works [278–287] revealed that interfaces between perfluorocarbons and aqueous media are usable for the research of morphological changes, division, and the viability of cells. Minami et al. successfully demonstrated the regulation of myogenic differentiation of C2C12 myoblast cells at water–perfluorocarbon interfaces as fully fluidic microenvironments. While the expression of MyoD remained at the usual level, the expression of myogenic regulatory factors was remarkably attenuated. The observed unusual regulation of myogenic differentiation was attributed to the fluidic nature of the water–perfluorocarbon interfaces. These interfacial cell culture systems might provide good opportunities to study mechanobiological effects in cell science and tissue engineering.

**Figure 26:**
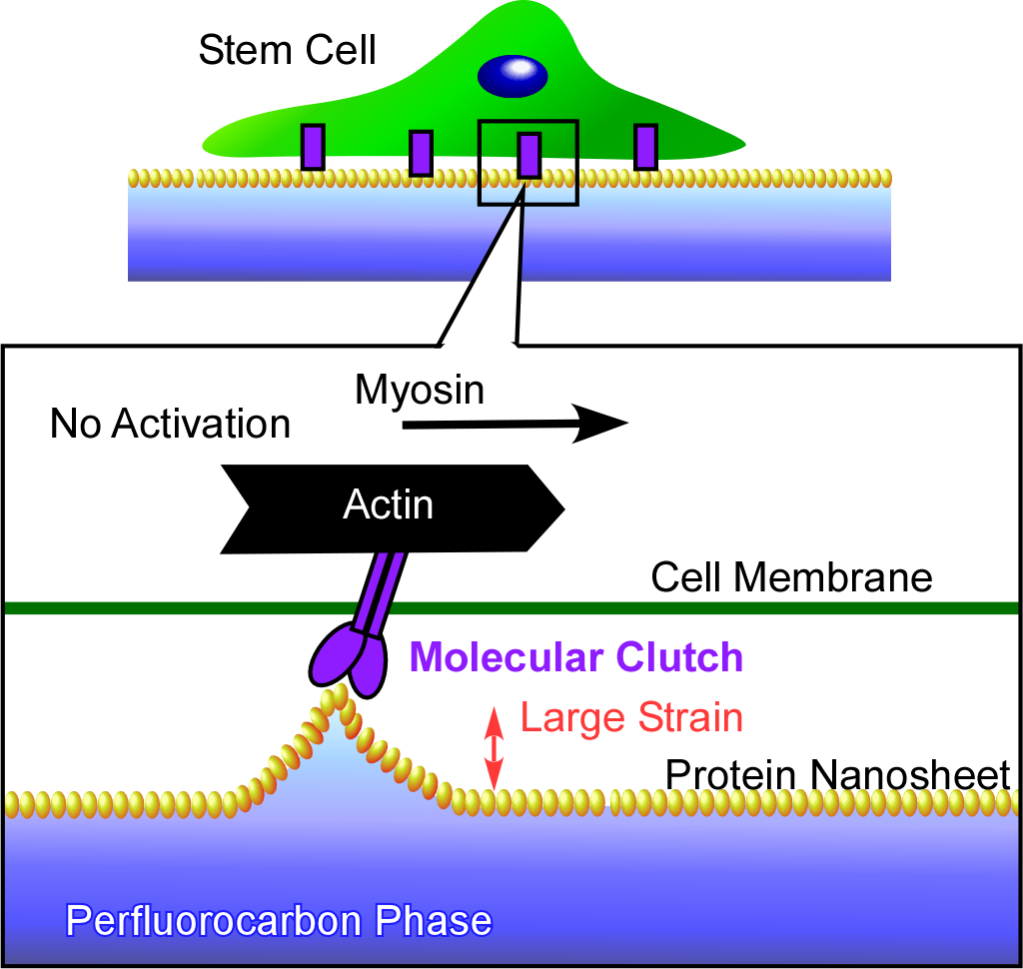
Human mesenchymal stem cells at the interface between aqueous medium and fluorocarbon phase with self-grown two-dimensional protein nanosheets at which the mechanical behaviour of cells can be explained by a molecular clutch model.

The formation of two-dimensional protein nanosheets and the modulation of stem cell mechanosensing at water–perfluorocarbon interfaces has been recently reported by Jia and co-workers [288]. At the water–perfluorocarbon interfaces, serum proteins were denatured to self-assemble into two-dimensional protein nanosheets. Their packing can be tuned through the selection of the perfluorocarbon compound, e.g., perfluorodecalin or perfluorotributylamine. Human mesenchymal stem cells are mechanically affected by the contact with the two-dimensional protein nanosheets. Spreading, adhesion growth, and yes-associated protein nuclear translocation of the cells were triggered by a greater stiffness of the two-dimensional protein nanosheets. The observed behaviour can be explained by a molecular clutch model (Figure 26). The underlying two-dimensional protein nanosheets are extremely flexible and exhibit a large strain upon traction by the cells, which is probably greater than the effect from solid surfaces. The obtained results are useful for the understanding of interactions between cells and materials at liquid interfacial media, as well as in the development of stem cell culture media, and regenerative therapies.

Yang and co-workers also demonstrated the fabrication of two-dimensional amyloid-like ultrathin two-dimensional protein membranes at the air–water interface [289]. The membranes are formed through fast aggregation of amyloid-like lysozyme molecules with controllable thickness from 30 to 250 nm accompanied by the formation of pores with diameters of 1.8 to 3.2 nm. While the two-dimensional membranes allow for a rather fast permeation of small substances, molecules and particles larger than 3 nm are retained at the membrane. Therefore, these two-dimensional materials exhibited an excellent hemodialysis capability to remove uremic toxins of medium molecular weight. The fabricated two-dimensional protein materials might be applied in pressure-driven filtration, size-directed forward osmosis, and large-scale dialysis systems.

**Figure 27:**
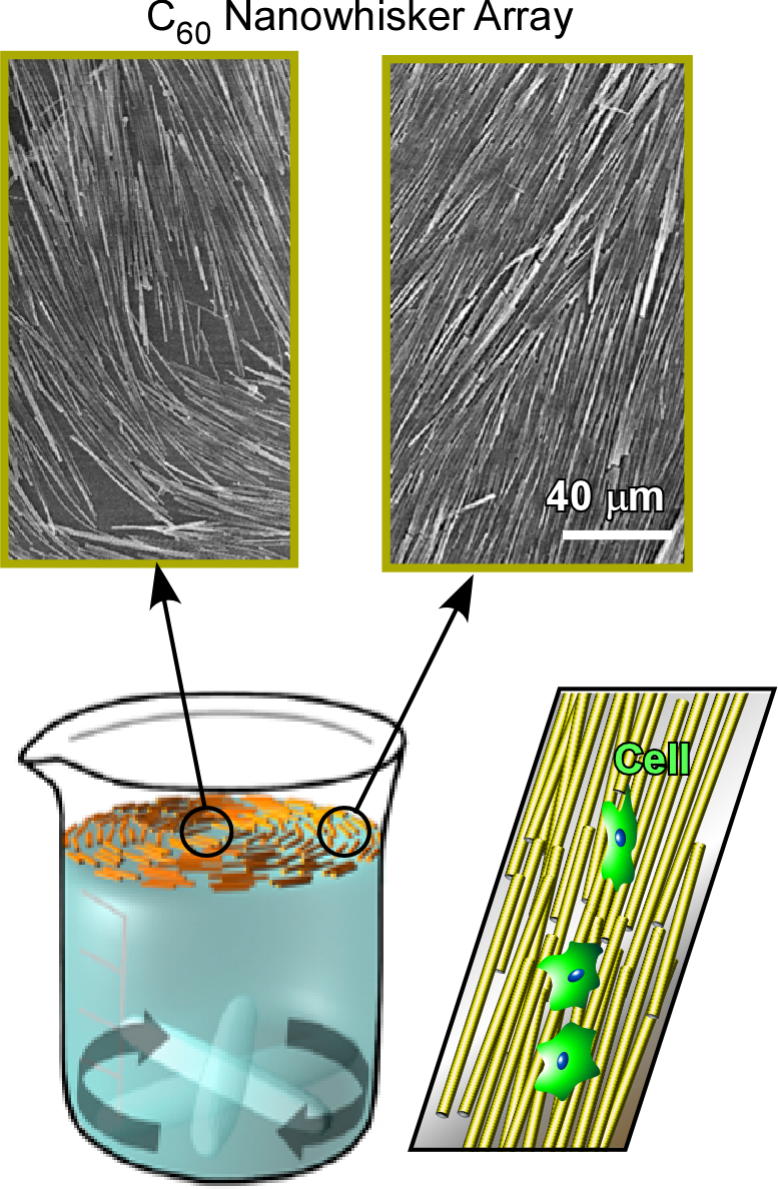
One-dimensional C_60_ nanowhiskers with controlled alignment and curvature fabricated by a vortex LB method for the culture of bone-forming human osteoblast MG63 cells.

Instead of spontaneously formed low-dimensional protein materials, low-dimensional fullerene materials artificially prepared at liquid interfaces have been used for the regulation of cell alignment and differentiation. Krishnan et al. applied a novel vortex LB method to align one-dimensional C_60_ nanowhiskers with controlled alignment and curvature (Figure 27) [290]. As described above, the vortex LB method utilizes the air–water interface with a vortex rotation of one subphase, which can align one-dimensional C_60_ nanowhiskers to the flow direction. The aligned nanowhiskers can be also transferred onto a solid substrate but their geometry can be regulated through the selection of the transfer position from the centre of vortex motion. While a transfer far from the centre resulted in almost parallel alignment, curved alignment can be obtained through transfer from near the vortex centre. The aligned C_60_ nanowhisker arrays were used as a scaffold for the culture of bone-forming human osteoblast MG63 cells. Cell growth occurred mostly along the axis of the aligned one-dimensional C_60_ nanowhiskers. In addition, the low toxicity of the C_60_ nanowhiskers was confirmed by cell proliferation test.

**Figure 28:**
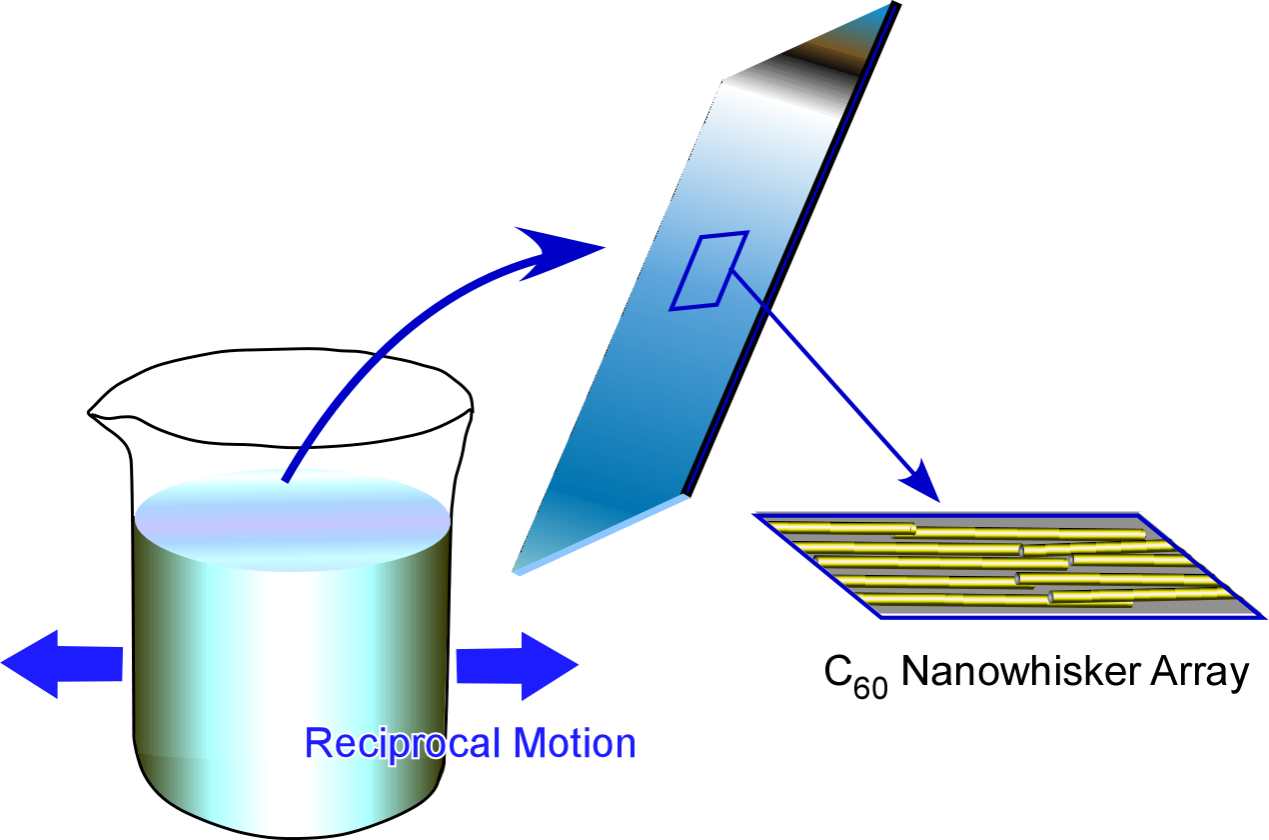
Highly aligned arrays of C_60_ nanowhiskers obtained by using a LB method with reciprocal motion in one direction for differentiation control of neural stem cells.

Minami et al. investigated the regulation of macroscopic cell orientation and differentiation of mouse skeletal myoblast C2C12 cells [291]. Highly aligned C_60_ nanowhisker arrays on a solid surface were fabricated via conventional LB transfer from the air–water interface. The culture of C2C12 cells on the aligned C_60_ nanowhiskers led to a significant enhancement of myotube formation with highly regulated directional growth. Hsu and co-workers proposed the fabrication of highly oriented and well-aligned arrays of one-dimensional C_60_ nanowhiskers through the modification of motion in a dynamic LB process [292]. Instead of vortex rotational motion, they used a reciprocal (shaking) motion in one direction for a monolayer of C_60_ nanowhiskers at the air–water interface, resulting in arrays of C_60_ nanowhiskers with a higher degree of alignment (Figure 28). The differentiation of neural stem cells on highly aligned C_60_ nanowhiskers was investigated to show their enhanced viability and their differentiation into mature neurons. Because of the biocompatibility of the used one-dimensional C_60_ nanowhiskers and the high potential for large-area fabrication, the presented C_60_ scaffolds might be a promising platform for patterned cell scaffolds for tissue engineering.

Highly expressible bacteriorhodopsin is known as a light-sensitive opsin with the potential capability of triggering neuronal activities through optogenetic modulation [293]. Optogenetic modulation and reprogramming of human fibroblasts transfected by highly expressible bacteriorhodopsin was investigated by Hsu and co-workers using tow-dimensional C_60_ nanosheets [294]. The transfected ﬁbroblasts cultured on C_60_ nanosheets with controlled light illumination induced reprogramming and differentiation into neural cells. This approach may indicate the possibility to control cell differentiation by selective location (on two-dimensional C_60_ nanosheets) and timing (illumination time).

### 6 Conclusion and Perspectives

For designing advanced functions within a small space, the selective and anisotropic organization of materials, energy, electrons, and information is indispensable. One of the promising methods to obtain such a unique aggregated state would be using low-dimensional anisotropic systems and materials. Low-dimensional materials including nanoparticle, nanorods, and nanosheets have been paid much attention in science and technology in this context recently. In addition to conventional low-dimensional materials such as fullerene, carbon nanotubes, graphene and various two-dimensional materials, constructing novel low-dimensional materials from a wide range of nanomaterials precursors is an attractive research field. A novel concept called nanoarchitectonics in which functional materials are engineered from nanoscale components, might be the best methodology for this objective. Especially, the use of dynamic interfacial media providing a unique restriction the molecular motion is advantageous for the facile synthesis of anisotropic low-dimensional materials.

Based on these general considerations, this review described bottom-up syntheses of low-dimensional systems and materials using materials nanoarchitectonics at two-dimensional liquid interfaces. The contents of the review article range from the description of basic characteristics of interfacial media with specific features of molecular interactions to various materials systems including molecular patterns, two-dimensional MOF/COF, low-dimensional nanocarbons, and biomaterial assemblies including living cells at liquid interfaces. Methodologies to realize materials nanoarchitectonics at two-dimensional liquid interfaces make use of various aspects and functions of low-dimensional systems and materials including the control of fundamental molecular interactions that regulate complicated cell functions. A generality of nanoarchitectonics concepts at liquid interfaces for low-dimensional systems and materials can be found in the examples introduced in this review.

For future developments of the nanoarchitectonics strategy, two essential features need to be considered in more detail. The first is the control of functional component units within low-dimensional materials, especially in two-dimensional planes. Very advanced functions seen in biological systems such as energy conversion and signal transduction highly rely on sophisticated arrangements and organizations of functional elements within two-dimensional cell membranes. These sophisticated biological systems need to be studied more extensively as an ideal example of low-dimensional functional materials. The regulation of the organization of functional components within low-dimensional structures would be a key methodology to develop low-dimensional systems to the next stage. Another key requirement of nanoarchitectonics would be the development of large-scale production. Most of research efforts on nanoarchitectonics-based low-dimensional materials are still at the laboratory scale, which is not attractive for further industrial developments. The use of low-cost precursors and application examples that can be realized with simple technical procedures would be important. Upon technical development according to these required features, low-dimensional systems and materials might give more opportunities to design advanced functions for industrial applications.

## References

[1] Silvestre B S, Tîrcă D M (2019). J Cleaner Prod.

[2] Povie G, Segawa Y, Nishihara T, Miyauchi Y, Itami K (2017). Science.

[3] Wang Y, Michinobu T (2017). Bull Chem Soc Jpn.

[4] Takimiya K, Nakano M (2018). Bull Chem Soc Jpn.

[5] Sun Z, Matsuno T, Isobe H (2018). Bull Chem Soc Jpn.

[6] Sun Z, Ikemoto K, Fukunaga T M, Koretsune T, Arita R, Sato S, Isobe H (2019). Science.

[7] Soloviev I I, Klenov N V, Bakurskiy S V, Kupriyanov M Y, Gudkov A L, Sidorenko A S (2017). Beilstein J Nanotechnol.

[8] Taniguchi M (2017). Bull Chem Soc Jpn.

[9] Su V-C, Chu C H, Sun G, Tsai D P (2018). Opt Express.

[10] Huang X, Wang J, Li T, Wang J, Xu M, Yu W, El Abed A, Zhang X (2018). Beilstein J Nanotechnol.

[11] Nishizawa M (2018). Bull Chem Soc Jpn.

[12] Li J-F, Zhang Y-J, Ding S-Y, Panneerselvam R, Tian Z-Q (2017). Chem Rev.

[13] Mizutani Y (2017). Bull Chem Soc Jpn.

[14] Zrimsek A B, Chiang N, Mattei M, Zaleski S, McAnally M O, Chapman C T, Henry A-I, Schatz G C, Van Duyne R P (2017). Chem Rev.

[15] Ruiz-Hitzky E, Gómez-Avilés A, Darder M, Aranda P (2018). Bull Chem Soc Jpn.

[16] Einaga Y (2018). Bull Chem Soc Jpn.

[17] Hu M, Reboul J, Furukawa S, Torad N L, Ji Q, Srinivasu P, Ariga K, Kitagawa S, Yamauchi Y (2012). J Am Chem Soc.

[18] Chaikittisilp W, Torad N L, Li C, Imura M, Suzuki N, Ishihara S, Ariga K, Yamauchi Y (2014). Chem – Eur J.

[19] Malgras V, Ji Q, Kamachi Y, Mori T, Shieh F-K, Wu K C-W, Ariga K, Yamauchi Y (2015). Bull Chem Soc Jpn.

[20] Wei Q, Xiong F, Tan S, Huang L, Lan E H, Dunn B, Mai L (2017). Adv Mater (Weinheim, Ger).

[21] Gon M, Tanaka K, Chujo Y (2017). Bull Chem Soc Jpn.

[22] Jana A, Scheer E, Polarz S (2017). Beilstein J Nanotechnol.

[23] Yao Q, Yuan X, Chen T, Leong D T, Xie J (2018). Adv Mater (Weinheim, Ger).

[24] Kobayashi Y (2018). Bull Chem Soc Jpn.

[25] Chaikittisilp W, Hu M, Wang H, Huang H-S, Fujita T, Wu K C-W, Chen L-C, Yamauchi Y, Ariga K (2012). Chem Commun.

[26] Torad N L, Hu M, Ishihara S, Sukegawa H, Belik A A, Imura M, Ariga K, Sakka Y, Yamauchi Y (2014). Small.

[27] Guo D, Shibuya R, Akiba C, Saji S, Kondo T, Nakamura J (2016). Science.

[28] Kumar S, Kumar A, Bahuguna A, Sharma V, Krishnan V (2017). Beilstein J Nanotechnol.

[29] Stauss S, Honma I (2018). Bull Chem Soc Jpn.

[30] Miyasaka T (2018). Bull Chem Soc Jpn.

[31] Watanabe M, Dokko K, Ueno K, Thomas M L (2018). Bull Chem Soc Jpn.

[32] Egorova K S, Gordeev E G, Ananikov V P (2017). Chem Rev.

[33] Saptiama I, Kaneti Y V, Suzuki Y, Suzuki Y, Tsuchiya K, Sakae T, Takai K, Fukumitsu N, Alothman Z A, Hossain M S A (2017). Bull Chem Soc Jpn.

[34] Chou K-C (2017). Curr Top Med Chem.

[35] Asanuma H, Murayama K, Kamiya Y, Kashida H (2018). Bull Chem Soc Jpn.

[36] Zheng K, Setyawati M I, Leong D T, Xie J (2018). Coord Chem Rev.

[37] Saptiama I, Kaneti Y V, Oveisi H, Suzuki Y, Tsuchiya K, Takai K, Sakae T, Pradhan S, Hossain M S A, Fukumitsu N (2018). Bull Chem Soc Jpn.

[38] Kiguchi M, Fujii S (2017). Bull Chem Soc Jpn.

[39] Yamamura A, Watanabe S, Uno M, Mitani M, Mitsui C, Tsurumi J, Isahaya N, Kanaoka Y, Okamoto T, Takeya J (2018). Sci Adv.

[40] Acharya S, Hill J P, Ariga K (2009). Adv Mater (Weinheim, Ger).

[41] Wang R, Lu K-Q, Tang Z-R, Xu Y-J (2017). J Mater Chem A.

[42] Lodahl P, Mahmoodian S, Stobbe S, Rauschenbeutel A, Schneeweiss P, Volz J, Pichler H, Zoller P (2017). Nature.

[43] Nakano K, Honda T, Yamasaki K, Tanaka Y, Taniguchi K, Ishimatsu R, Imato T (2018). Bull Chem Soc Jpn.

[44] Ding X, Peng F, Zhou J, Gong W, Slaven G, Loh K P, Lim C T, Leong D T (2019). Nat Commun.

[45] Datta K K R, Reddy B V S, Ariga K, Vinu A (2010). Angew Chem, Int Ed.

[46] Shirai H, Nguyen M T, Čempel D, Tsukamoto H, Tokunaga T, Liao Y-C, Yonezawa T (2017). Bull Chem Soc Jpn.

[47] Huang R, Chen H, Xia Z (2017). Bull Chem Soc Jpn.

[48] Choukourov A, Pleskunov P, Nikitin D, Titov V, Shelemin A, Vaidulych M, Kuzminova A, Solař P, Hanuš J, Kousal J (2017). Beilstein J Nanotechnol.

[49] Bhattacharyya K, Mukherjee S (2018). Bull Chem Soc Jpn.

[50] Kani K, Zakaria M B, Lin J, Alshehri A A, Kim J, Bando Y, You J, Hossain M S A, Bo J, Yamauchi Y (2018). Bull Chem Soc Jpn.

[51] Yonezawa T, Čempel D, Nguyen M T (2018). Bull Chem Soc Jpn.

[52] Zhang R, Zhang Y, Wei F (2017). Chem Soc Rev.

[53] Kharlamova M V (2017). Beilstein J Nanotechnol.

[54] Clancy A J, Anthony D B, Fisher S J, Leese H S, Roberts C S, Shaffer M S P (2017). Nanoscale.

[55] Chen R, Kang J, Kang M, Lee H, Lee H (2018). Bull Chem Soc Jpn.

[56] Ji Q, Honma I, Paek S-M, Akada M, Hill J P, Vinu A, Ariga K (2010). Angew Chem, Int Ed.

[57] Anasori B, Lukatskaya M R, Gogotsi Y (2017). Nat Rev Mater.

[58] Khan A H, Ghosh S, Pradhan B, Dalui A, Shrestha L K, Acharya S, Ariga K (2017). Bull Chem Soc Jpn.

[59] Manzeli S, Ovchinnikov D, Pasquier D, Yazyev O V, Kis A (2017). Nat Rev Mater.

[60] Ariga K, Watanabe S, Mori T, Takeya J (2018). NPG Asia Mater.

[61] Maeda K, Mallouk T E (2019). Bull Chem Soc Jpn.

[62] Ulaganathan R K, Chang Y-H, Wang D-Y, Li S-S (2018). Bull Chem Soc Jpn.

[63] Sakamoto R (2017). Bull Chem Soc Jpn.

[64] Liu J, Chen Q-W, Wu K (2017). Chin Chem Lett.

[65] Ariga K, Mori T, Shrestha L K (2018). Chem Rec.

[66] Cao L, Wang T, Wang C (2018). Chin J Chem.

[67] Wang Y, Mayorga-Martinez C C, Pumera M (2017). Bull Chem Soc Jpn.

[68] Ariga K, Jia X, Shrestha L K (2019). Mol Syst Des Eng.

[69] Seki T (2018). Bull Chem Soc Jpn.

[70] Vinu A, Miyahara M, Ariga K (2006). J Nanosci Nanotechnol.

[71] Katagiri K, Hashizume M, Ariga K, Terashima T, Kikuchi J-i (2007). Chem – Eur J.

[72] Ariga K, Hill J P, Lee M V, Vinu A, Charvet R, Acharya S (2008). Sci Technol Adv Mater.

[73] Ariga K, Hill J P, Ji Q (2008). Macromol Biosci.

[74] Haketa Y, Maeda H (2018). Bull Chem Soc Jpn.

[75] Ariga K, Nishikawa M, Mori T, Takeya J, Shrestha L K, Hill J P (2019). Sci Technol Adv Mater.

[76] Hiraoka S (2018). Bull Chem Soc Jpn.

[77] Dhiman S, George S J (2018). Bull Chem Soc Jpn.

[78] Shimizu T (2018). Bull Chem Soc Jpn.

[79] Komiyama M, Mori T, Ariga K (2018). Bull Chem Soc Jpn.

[80] Akamatsu M, Komatsu H, Matsuda A, Mori T, Nakanishi W, Sakai H, Hill J P, Ariga K (2017). Bull Chem Soc Jpn.

[81] Suda M (2018). Bull Chem Soc Jpn.

[82] Abdullayev E, Sakakibara K, Okamoto K, Wei W, Ariga K, Lvov Y (2011). ACS Appl Mater Interfaces.

[83] Vinokurov V A, Stavitskaya A V, Chudakov Y A, Ivanov E V, Shrestha L K, Ariga K, Darrat Y A, Lvov Y M (2017). Sci Technol Adv Mater.

[84] Glotov A, Stavitskaya A, Chudakov Y, Ivanov E, Huang W, Vinokurov V, Zolotukhina A, Maximov A, Karakhanov E, Lvov Y (2019). Bull Chem Soc Jpn.

[85] Zhong S, Xu Q (2018). Bull Chem Soc Jpn.

[86] Sengottaiyan C, Jayavel R, Bairi P, Shrestha R G, Ariga K, Shrestha L K (2017). Bull Chem Soc Jpn.

[87] Li B L, Setyawati M I, Chen L, Xie J, Ariga K, Lim C-T, Garaj S, Leong D T (2017). ACS Appl Mater Interfaces.

[88] Ariga K, Ji Q, Hill J P, Bando Y, Aono M (2012). NPG Asia Mater.

[89] Ariga K, Ji Q, Nakanishi W, Hill J P, Aono M (2015). Mater Horiz.

[90] Ariga K, Yamauchi Y, Aono M (2015). APL Mater.

[91] Ariga K, Aono M (2016). Jpn J Appl Phys.

[92] Ariga K, Li M, Richards G, Hill J (2011). J Nanosci Nanotechnol.

[93] Ariga K, Li J, Fei J, Ji Q, Hill J P (2016). Adv Mater (Weinheim, Ger).

[94] Shirai Y, Minami K, Nakanishi W, Yonamine Y, Joachim C, Ariga K (2016). Jpn J Appl Phys.

[95] Aono M, Ariga K (2016). Adv Mater (Weinheim, Ger).

[96] Ariga K (2017). Mater Chem Front.

[97] Ariga K, Vinu A, Yamauchi Y, Ji Q, Hill J P (2012). Bull Chem Soc Jpn.

[98] Govindaraju T, Avinash M B (2012). Nanoscale.

[99] Ramanathan M, Shrestha L K, Mori T, Ji Q, Hill J P, Ariga K (2013). Phys Chem Chem Phys.

[100] Zerkoune L, Angelova A, Lesieur S (2014). Nanomaterials.

[101] Ariga K, Ji Q, Nakanishi W, Hill J P (2015). J Inorg Organomet Polym Mater.

[102] Shrestha L K, Strzelczyk K M, Shrestha R G, Ichikawa K, Aramaki K, Hill J P, Ariga K (2015). Nanotechnology.

[103] Hecht S (2003). Angew Chem, Int Ed.

[104] Ariga K, Lee M V, Mori T, Yu X-Y, Hill J P (2010). Adv Colloid Interface Sci.

[105] Ramanathan M, Hong K, Ji Q, Yonamine Y, Hill J P, Ariga K (2014). J Nanosci Nanotechnol.

[106] Wakayama Y (2016). Jpn J Appl Phys.

[107] Ariga K, Malgras V, Ji Q, Zakaria M B, Yamauchi Y (2016). Coord Chem Rev.

[108] Sangian D, Ide Y, Bando Y, Rowan A E, Yamauchi Y (2018). Small.

[109] Ishihara S, Labuta J, Van Rossom W, Ishikawa D, Minami K, Hill J P, Ariga K (2014). Phys Chem Chem Phys.

[110] Ariga K, Yamauchi Y, Ji Q, Yonamine Y, Hill J P (2014). APL Mater.

[111] Ariga K, Minami K, Shrestha L K (2016). Analyst.

[112] Jackman J A, Cho N-J, Nishikawa M, Yoshikawa G, Mori T, Shrestha L K, Ariga K (2018). Chem – Asian J.

[113] Rajendran R, Shrestha L K, Minami K, Subramanian M, Jayavel R, Ariga K (2014). J Mater Chem A.

[114] Rajendran R, Shrestha L K, Kumar R M, Jayavel R, Hill J P, Ariga K (2015). J Inorg Organomet Polym Mater.

[115] Chen R, Zhao T, Zhang X, Li L, Wu F (2016). Nanoscale Horiz.

[116] Kim J, Kim J H, Ariga K (2017). Joule.

[117] Shrestha L K, Shrestha R G, Joshi S, Rajbhandari R, Shrestha N, Adhikari M P, Pradhananga R R, Ariga K (2017). J Inorg Organomet Polym Mater.

[118] Ariga K, Ishihara S, Abe H, Li M, Hill J P (2012). J Mater Chem.

[119] Puscasu C M, Carja G, Zaharia C (2015). Int J Mater Prod Technol.

[120] Ariga K, Ishihara S, Abe H (2016). CrystEngComm.

[121] Wang H, Yin S, Eid K, Li Y, Xu Y, Li X, Xue H, Wang L (2018). ACS Sustainable Chem Eng.

[122] Ariga K, Ji Q, Mori T, Naito M, Yamauchi Y, Abe H, Hill J P (2013). Chem Soc Rev.

[123] Nakanishi W, Minami K, Shrestha L K, Ji Q, Hill J P, Ariga K (2014). Nano Today.

[124] Ariga K, Kawakami K, Ebara M, Kotsuchibashi Y, Ji Q, Hill J P (2014). New J Chem.

[125] Pandey A P, Girase N M, Patil M D, Patil P O, Patil D A, Deshmukh P K (2014). J Nanosci Nanotechnol.

[126] Ariga K, Naito M, Ji Q, Payra D (2016). CrystEngComm.

[127] Nayak A, Unayama S, Tai S, Tsuruoka T, Waser R, Aono M, Valov I, Hasegawa T (2018). Adv Mater (Weinheim, Ger).

[128] Yan Y, Ye J, Wang K, Yao J, Zhao Y S (2018). Small.

[129] Stulz E (2017). Acc Chem Res.

[130] Pandeeswar M, Senanayak S P, Govindaraju T (2016). ACS Appl Mater Interfaces.

[131] Zhang L, Wang T, Shen Z, Liu M (2016). Adv Mater (Weinheim, Ger).

[132] Zhao L, Zou Q, Yan X (2019). Bull Chem Soc Jpn.

[133] Abe H, Liu J, Ariga K (2016). Mater Today.

[134] Osada M, Sasaki T (2018). Dalton Trans.

[135] Xu J, Zhang J, Zhang W, Lee C-S (2017). Adv Energy Mater.

[136] Mathesh M, Liu J, Barrow C J, Yang W (2017). Chem – Eur J.

[137] Ariga K, Yamauchi Y, Mori T, Hill J P (2013). Adv Mater (Weinheim, Ger).

[138] Ariga K, Mori T, Nakanishi W (2018). Chem – Asian J.

[139] Ariga K, Ishihara S, Izawa H, Xia H, Hill J P (2011). Phys Chem Chem Phys.

[140] Ariga K, Hill J P (2011). Chem Rec.

[141] Ariga K, Mori T, Hill J P (2012). Soft Matter.

[142] Ariga K, Kunitake T (1998). Acc Chem Res.

[143] Ariga K, Ito H, Hill J P, Tsukube H (2012). Chem Soc Rev.

[144] Ariga K, Mori T, Li J (2019). Langmuir.

[145] Kurihara K, Ohto K, Tanaka Y, Aoyama Y, Kunitake T (1989). Thin Solid Films.

[146] Kurihara K, Ohto K, Tanaka Y, Aoyama Y, Kunitake T (1991). J Am Chem Soc.

[147] Cha X, Ariga K, Onda M, Kunitake T (1995). J Am Chem Soc.

[148] Cha X, Ariga K, Kunitake T (1996). J Am Chem Soc.

[149] Ariga K, Kamino A, Cha X, Kunitake T (1999). Langmuir.

[150] Ikeura Y, Kurihara K, Kunitake T (1991). J Am Chem Soc.

[151] Kurihara K, Ohto K, Honda Y, Kunitake T (1991). J Am Chem Soc.

[152] Kawahara T, Kurihara K, Kunitake T (1992). Chem Lett.

[153] Taguchi K, Ariga K, Kunitake T (1995). Chem Lett.

[154] Ariga K, Kamino A, Koyano H, Kunitake T (1997). J Mater Chem.

[155] Onda M, Yoshihara K, Koyano H, Ariga K, Kunitake T (1996). J Am Chem Soc.

[156] Springs B, Haake P (1977). Bioorg Chem.

[157] Sasaki D Y, Kurihara K, Kunitake T (1991). J Am Chem Soc.

[158] Sasaki D Y, Kurihara K, Kunitake T (1992). J Am Chem Soc.

[159] Sakurai M, Tamagawa H, Furuki T, Inoue Y, Ariga K, Kunitake T (1995). Chem Lett.

[160] Sakurai M, Tamagawa H, Inoue Y, Ariga K, Kunitake T (1997). J Phys Chem B.

[161] Tamagawa H, Sakurai M, Inoue Y, Ariga K, Kunitake T (1997). J Phys Chem B.

[162] Ariga K (2016). ChemNanoMat.

[163] Ariga K, Mori T, Hill J P (2012). Adv Mater (Weinheim, Ger).

[164] Ariga K, Mori T, Hill J P (2013). Langmuir.

[165] Ariga K, Mori T, Ishihara S, Kawakami K, Hill J P (2014). Chem Mater.

[166] Ariga K (2016). Anal Sci.

[167] Ariga K, Terasaka Y, Sakai D, Tsuji H, Kikuchi J-i (2000). J Am Chem Soc.

[168] Ariga K, Nakanishi T, Terasaka Y, Tsuji H, Sakai D, Kikuchi J-i (2005). Langmuir.

[169] Mori T, Komatsu H, Sakamoto N, Suzuki K, Hill J P, Matsumoto M, Sakai H, Ariga K, Nakanishi W (2018). Phys Chem Chem Phys.

[170] Mori T, Chin H, Kawashima K, Ngo H, Cho N-J, Nakanishi W, Hill J P, Ariga K (2019). ACS Nano.

[171] Ishikawa D, Mori T, Yonamine Y, Nakanishi W, Cheung D L, Hill J P, Ariga K (2015). Angew Chem, Int Ed.

[172] Mori T, Ishikawa D, Yonamine Y, Fujii Y, Hill J P, Ichinose I, Ariga K, Nakanishi W (2017). ChemPhysChem.

[173] Sakakibara K, Joyce L A, Mori T, Fujisawa T, Shabbir S H, Hill J P, Anslyn E V, Ariga K (2012). Angew Chem, Int Ed.

[174] Michinobu T, Shinoda S, Nakanishi T, Hill J P, Fujii K, Player T N, Tsukube H, Ariga K (2006). J Am Chem Soc.

[175] Michinobu T, Shinoda S, Nakanishi T, Hill J P, Fujii K, Player T N, Tsukube H, Ariga K (2011). Phys Chem Chem Phys.

[176] Mori T, Okamoto K, Endo H, Hill J P, Shinoda S, Matsukura M, Tsukube H, Suzuki Y, Kanekiyo Y, Ariga K (2010). J Am Chem Soc.

[177] Mori T, Okamoto K, Endo H, Sakakibara K, Hill J P, Shinoda S, Matsukura M, Tsukube H, Suzuki Y, Kanekiyo Y (2011). Nanoscale Res Lett.

[178] Ariga K, Minami K, Ebara M, Nakanishi J (2016). Polym J.

[179] Ariga K, Mori T, Nakanishi W, Hill J P (2017). Phys Chem Chem Phys.

[180] Shrestha L K, Mori T, Ariga K (2018). Curr Opin Colloid Interface Sci.

[181] Lehn J-M (1988). Angew Chem, Int Ed Engl.

[182] Cram D J (1988). Angew Chem, Int Ed Engl.

[183] Pedersen C J (1988). Angew Chem, Int Ed Engl.

[184] Shinkai S, Ogawa T, Nakaji T, Kusano Y, Nanabe O (1979). Tetrahedron Lett.

[185] Ueda A (2017). Bull Chem Soc Jpn.

[186] Gropp C, Quigley B L, Diederich F (2018). J Am Chem Soc.

[187] Irie M, Morimoto M (2018). Bull Chem Soc Jpn.

[188] Park J S, Sessler J L (2018). Acc Chem Res.

[189] Stoddart J F (2017). Angew Chem, Int Ed.

[190] Feringa B L (2017). Angew Chem, Int Ed.

[191] Sauvage J-P (2017). Angew Chem, Int Ed.

[192] Ariga K (2004). J Nanosci Nanotechnol.

[193] Ariga K (2008). J Photopolym Sci Technol.

[194] Oishi Y, Torii Y, Kuramori M, Suehiro K, Ariga K, Taguchi K, Kamino A, Kunitake T (1996). Chem Lett.

[195] Oishi Y, Torii Y, Kato T, Kuramori M, Suehiro K, Ariga K, Taguchi K, Kamino A, Koyano H, Kunitake T (1997). Langmuir.

[196] Oishi Y, Kato T, Kuramori M, Suehiro K, Ariga K, Kamino A, Koyano H, Kunitake T (1997). Chem Commun.

[197] Koyano H, Yoshihara K, Ariga K, Kunitake T, Oishi Y, Kawano O, Kuramori M, Suehiro K (1996). Chem Commun.

[198] Oishi Y, Kato T, Narita T, Ariga K, Kunitake T (2008). Langmuir.

[199] Liu X, Riess J G, Krafft M P (2018). Bull Chem Soc Jpn.

[200] Richard-Lacroix M, Borozenko K, Pellerin C, Bazuin C G (2016). Macromolecules.

[201] Wu T, Wen G, Huang C (2016). J Polym Sci, Part B: Polym Phys.

[202] Mori T, Sakakibara K, Endo H, Akada M, Okamoto K, Shundo A, Lee M V, Ji Q, Fujisawa T, Oka K (2013). Langmuir.

[203] Das T, Häring M, Haldar D, Díaz Díaz D (2018). Biomater Sci.

[204] Hanabusa K, Nakashima M, Funatsu E, Kishi S, Suzuki M (2018). Bull Chem Soc Jpn.

[205] Ganta S, Chand D K (2018). Chem – Asian J.

[206] Sasaki J, Suzuki M, Hanabusa K (2018). Bull Chem Soc Jpn.

[207] Cherumukkil S, Vedhanarayanan B, Das G, Praveen V K, Ajayaghosh A (2018). Bull Chem Soc Jpn.

[208] Sakakibara K, Chithra P, Das B, Mori T, Akada M, Labuta J, Tsuruoka T, Maji S, Furumi S, Shrestha L K (2014). J Am Chem Soc.

[209] Vinu A, Srinivasu P, Sawant D P, Mori T, Ariga K, Chang J-S, Jhung S-H, Balasubramanian V V, Hwang Y K (2007). Chem Mater.

[210] Ariga K, Ji Q, McShane M J, Lvov Y M, Vinu A, Hill J P (2012). Chem Mater.

[211] Chaikittisilp W, Ariga K, Yamauchi Y (2013). J Mater Chem A.

[212] Zhang J, Yang X, Deng H, Qiao K, Farooq U, Ishaq M, Yi F, Liu H, Tang J, Song H (2017). Nano-Micro Lett.

[213] Jeevanandam J, Barhoum A, Chan Y S, Dufresne A, Danquah M K (2018). Beilstein J Nanotechnol.

[214] Smith M D, Karunadasa H I (2018). Acc Chem Res.

[215] Ogawa S, Wakayama T, Watanabe H, Hayashi K, Ogata S, Oaki Y, Hasegawa M, Imai H (2018). Bull Chem Soc Jpn.

[216] Cheng W, Ju Y, Payamyar P, Primc D, Rao J, Willa C, Koziej D, Niederberger M (2015). Angew Chem, Int Ed.

[217] Tiu B D B, Pernites R B, Foster E L, Advincula R C (2015). J Colloid Interface Sci.

[218] Nie H-L, Dou X, Tang Z, Jang H D, Huang J (2015). J Am Chem Soc.

[219] Yonamine Y, Cervantes-Salguero K, Minami K, Kawamata I, Nakanishi W, Hill J P, Murata S, Ariga K (2016). Phys Chem Chem Phys.

[220] Wang L, Sahabudeen H, Zhang T, Dong R (2018). npj 2D Mater Appl.

[221] Culp J T, Park J-H, Stratakis D, Meisel M W, Talham D R (2002). J Am Chem Soc.

[222] Makiura R, Motoyama S, Umemura Y, Yamanaka H, Sakata O, Kitagawa H (2010). Nat Mater.

[223] Wu G, Huang J, Zang Y, He J, Xu G (2017). J Am Chem Soc.

[224] Feldblyum J I, McCreery C H, Andrews S C, Kurosawa T, Santos E J G, Duong V, Fang L, Ayzner A L, Bao Z (2015). Chem Commun.

[225] Dai W, Shao F, Szczerbiński J, McCaffrey R, Zenobi R, Jin Y, Schlüter A D, Zhang W (2016). Angew Chem, Int Ed.

[226] Kambe T, Sakamoto R, Hoshiko K, Takada K, Miyachi M, Ryu J-H, Sasaki S, Kim J, Nakazato K, Takata M (2013). J Am Chem Soc.

[227] Takada K, Sakamoto R, Yi S-T, Katagiri S, Kambe T, Nishihara H (2015). J Am Chem Soc.

[228] Sahabudeen H, Qi H, Glatz B A, Tranca D, Dong R, Hou Y, Zhang T, Kuttner C, Lehnert T, Seifert G (2016). Nat Commun.

[229] Dey K, Pal M, Rout K C, Kunjattu H S, Das A, Mukherjee R, Kharul U K, Banerjee R (2017). J Am Chem Soc.

[230] Matsumoto M, Valentino L, Stiehl G M, Balch H B, Corcos A R, Wang F, Ralph D C, Mariñas B J, Dichtel W R (2018). Chem.

[231] Matsumoto M, Dasari R R, Ji W, Feriante C H, Parker T C, Marder S R, Dichtel W R (2017). J Am Chem Soc.

[232] Valentino L, Matsumoto M, Dichtel W R, Mariñas B J (2017). Environ Sci Technol.

[233] Sarikhani Z, Manoochehri M (2017). Bull Chem Soc Jpn.

[234] Mori T, Tanaka H, Dalui A, Mitoma N, Suzuki K, Matsumoto M, Aggarwal N, Patnaik A, Acharya S, Shrestha L K (2018). Angew Chem, Int Ed.

[235] Nakanishi T, Michinobu T, Yoshida K, Shirahata N, Ariga K, Möhwald H, Kurth D G (2008). Adv Mater (Weinheim, Ger).

[236] Shrestha L K, Shrestha R G, Hill J P, Ariga K (2013). J Oleo Sci.

[237] Miyazawa K, Kuwasaki Y, Hamamoto K, Nagata S, Obayashi A, Kuwabara M (2003). Surf Interface Anal.

[238] Shrestha L K, Ji Q, Mori T, Miyazawa K, Yamauchi Y, Hill J P, Ariga K (2013). Chem – Asian J.

[239] Miyazawa K (2015). Sci Technol Adv Mater.

[240] Shrestha L K, Hill J P, Miyazawa K, Ariga K (2012). J Nanosci Nanotechnol.

[241] Shrestha L K, Hill J P, Tsuruoka T, Miyazawa K, Ariga K (2013). Langmuir.

[242] Shrestha L K, Shrestha R G, Yamauchi Y, Hill J P, Nishimura T, Miyazawa K, Kawai T, Okada S, Wakabayashi K, Ariga K (2015). Angew Chem, Int Ed.

[243] Bairi P, Shrestha R G, Hill J P, Nishimura T, Ariga K, Shrestha L K (2016). J Mater Chem A.

[244] Shrestha R G, Shrestha L K, Khan A H, Kumar G S, Acharya S, Ariga K (2014). ACS Appl Mater Interfaces.

[245] Tang Q, Zhang S, Liu X, Sumita M, Ishihara S, Fuchs H, Ji Q, Shrestha L K, Ariga K (2017). Phys Chem Chem Phys.

[246] Kumar G S, Shrestha R G, Ji Q, Hill J P, Ariga K, Acharya S, Shrestha L K (2018). Phys Chem Chem Phys.

[247] Furuuchi N, Shrestha R, Yamashita Y, Hirao T, Ariga K, Shrestha L (2019). Sensors.

[248] Saran R, Curry R J (2018). Small.

[249] Shen W, Zhang L, Zheng S, Xie Y, Lu X (2017). ACS Appl Mater Interfaces.

[250] Shrestha L K, Yamauchi Y, Hill J P, Miyazawa K, Ariga K (2013). J Am Chem Soc.

[251] Tang Q, Bairi P, Shrestha R G, Hill J P, Ariga K, Zeng H, Ji Q, Shrestha L K (2017). ACS Appl Mater Interfaces.

[252] Shrestha L K, Sathish M, Hill J P, Miyazawa K, Tsuruoka T, Sanchez-Ballester N M, Honma I, Ji Q, Ariga K (2013). J Mater Chem C.

[253] Bairi P, Minami K, Nakanishi W, Hill J P, Ariga K, Shrestha L K (2016). ACS Nano.

[254] Bairi P, Minami K, Hill J P, Ariga K, Shrestha L K (2017). ACS Nano.

[255] Bairi P, Tsuruoka T, Acharya S, Ji Q, Hill J P, Ariga K, Yamauchi Y, Shrestha L K (2018). Mater Horiz.

[256] Shrestha L K, Shrestha R G, Hill J P, Tsuruoka T, Ji Q, Nishimura T, Ariga K (2016). Langmuir.

[257] Wang B, Zheng S, Saha A, Bao L, Lu X, Guldi D M (2017). J Am Chem Soc.

[258] Zhou S, Wang L, Chen M, Liu B, Sun X, Cai M, Li H (2017). Nanoscale.

[259] Bairi P, Minami K, Hill J P, Nakanishi W, Shrestha L K, Liu C, Harano K, Nakamura E, Ariga K (2016). ACS Nano.

[260] Komiyama M, Yoshimoto K, Sisido M, Ariga K (2017). Bull Chem Soc Jpn.

[261] Ariga K, Leong D T, Mori T (2018). Adv Funct Mater.

[262] Ariga K, Jackman J A, Cho N-J, Hsu S-h, Shrestha L K, Mori T, Takeya J Chem Rec.

[263] Matsuura K (2017). Bull Chem Soc Jpn.

[264] Sawada T, Serizawa T (2018). Bull Chem Soc Jpn.

[265] He H, Xu B (2018). Bull Chem Soc Jpn.

[266] Fromherz P (1971). Biochim Biophys Acta, Biomembr.

[267] Fromherz P (1975). Rev Sci Instrum.

[268] Okahata Y, Tsuruta T, Ijiro K, Ariga K (1988). Langmuir.

[269] Okahata Y, Tsuruta T, Ijiro K, Ariga K (1989). Thin Solid Films.

[270] Ariga K, Hill J P, Ji Q (2007). Phys Chem Chem Phys.

[271] Ariga K, Lvov Y M, Kawakami K, Ji Q, Hill J P (2011). Adv Drug Delivery Rev.

[272] Ariga K, Yamauchi Y, Rydzek G, Ji Q, Yonamine Y, Wu K C-W, Hill J P (2014). Chem Lett.

[273] Rydzek G, Ji Q, Li M, Schaaf P, Hill J P, Boulmedais F, Ariga K (2015). Nano Today.

[274] Onda M, Lvov Y, Ariga K, Kunitake T (1996). J Ferment Bioeng.

[275] Onda M, Lvov Y, Ariga K, Kunitake T (1996). Biotechnol Bioeng.

[276] Onda M, Ariga K, Kunitake T (1999). J Biosci Bioeng.

[277] Minami K, Mori T, Nakanishi W, Shigi N, Nakanishi J, Hill J P, Komiyama M, Ariga K (2017). ACS Appl Mater Interfaces.

[278] Giaever I, Keese C R (1983). Proc Natl Acad Sci U S A.

[279] Keese C R, Giaever I (1983). Proc Natl Acad Sci U S A.

[280] Keese C R, Giaever I (1991). Exp Cell Res.

[281] Shiba Y, Ohshima T, Sato M (1998). Kagaku Kogaku Ronbunshu.

[282] Shiba Y, Ohshima T, Sato M (1998). Biotechnol Bioeng.

[283] Sato M, Shinozawa T, Ueno H, Sadakata M (1991). Kagaku Kogaku Ronbunshu.

[284] Ando J, Albelda S M, Levine E M (1991). In Vitro Cell Dev Biol: Anim.

[285] Pilarek M, Grabowska I, Ciemerych M A, Dąbkowska K, Szewczyk K W (2013). Biotechnol Lett.

[286] Kong D, Megone W, Nguyen K D Q, Di Cio S, Ramstedt M, Gautrot J E (2018). Nano Lett.

[287] Kong D, Peng L, Di Cio S, Novak P, Gautrot J E (2018). ACS Nano.

[288] Jia X, Minami K, Uto K, Chang A C, Hill J P, Ueki T, Nakanishi J, Ariga K (2019). Small.

[289] Yang F, Tao F, Li C, Gao L, Yang P (2018). Nat Commun.

[290] Krishnan V, Kasuya Y, Ji Q, Sathish M, Shrestha L K, Ishihara S, Minami K, Morita H, Yamazaki T, Hanagata N (2015). ACS Appl Mater Interfaces.

[291] Minami K, Kasuya Y, Yamazaki T, Ji Q, Nakanishi W, Hill J P, Sakai H, Ariga K (2015). Adv Mater (Weinheim, Ger).

[292] Hsieh F-Y, Shrestha L K, Ariga K, Hsu S-h (2017). Chem Commun.

[293] Deisseroth K (2011). Nat Methods.

[294] Luo P-W, Han H-W, Yang C-S, Shrestha L K, Ariga K, Hsu S-h (2019). Adv Biosyst.

